# Deep learning-based scoring of tumour-infiltrating lymphocytes is prognostic in primary melanoma and predictive to PD-1 checkpoint inhibition in melanoma metastases

**DOI:** 10.1016/j.ebiom.2023.104644

**Published:** 2023-06-07

**Authors:** Eftychia Chatziioannou, Jana Roßner, Thazin New Aung, David L. Rimm, Heike Niessner, Ulrike Keim, Lina Maria Serna-Higuita, Irina Bonzheim, Luis Kuhn Cuellar, Dana Westphal, Julian Steininger, Friedegund Meier, Oltin Tiberiu Pop, Stephan Forchhammer, Lukas Flatz, Thomas Eigentler, Claus Garbe, Martin Röcken, Teresa Amaral, Tobias Sinnberg

**Affiliations:** a Department of Dermatology, University of Tübingen, Liebermeisterstr. 25, 72076 Tübingen, Germany; b Cluster of Excellence iFIT (EXC 2180) “Image-Guided and Functionally Instructed Tumor Therapies”, Tübingen, Germany; c Department of Dermatology, University of Heidelberg, Im Neuenheimer Feld 440, 69120 Heidelberg, Germany; d Department of Pathology, Yale University School of Medicine, New Haven, CT, USA; e Department of Clinical Epidemiology and Applied Biostatistics, Eberhard Karls University of Tübingen, 72076 Tübingen, Germany; f Institute of Pathology and Neuropathology, Eberhard Karls University of Tübingen, 72076 Tübingen, Germany; g Quantitative Biology Center (QBiC), University of Tübingen, Tübingen, Germany; h Department of Dermatology, Faculty of Medicine and University Hospital Carl Gustav Carus, Skin Cancer Center at the University Cancer Center and National Center for Tumor Diseases, Technical University Dresden, 01307 Dresden, Germany; i Institute of Immunobiology, Kantonsspital St. Gallen, St. Gallen, Switzerland; j Department of Dermatology, Venereology and Allergology, Charité-Universitätsmedizin Berlin, Charitéplatz 1, 10117 Berlin, Germany

**Keywords:** Cutaneous melanoma, Prognostic biomarkers, Predictive biomarkers, Tumour-infiltrating lymphocytes, Digital pathology

## Abstract

**Background:**

Recent advances in digital pathology have enabled accurate and standardised enumeration of tumour-infiltrating lymphocytes (TILs). Here, we aim to evaluate TILs as a percentage electronic TIL score (eTILs) and investigate its prognostic and predictive relevance in cutaneous melanoma.

**Methods:**

We included stage I to IV cutaneous melanoma patients and used hematoxylin-eosin-stained slides for TIL analysis. We assessed eTILs as a continuous and categorical variable using the published cut-off of 16.6% and applied Cox regression models to evaluate associations of eTILs with relapse-free, distant metastasis-free, and overall survival. We compared eTILs of the primaries with matched metastasis. Moreover, we assessed the predictive relevance of eTILs in therapy-naïve metastases according to the first-line therapy.

**Findings:**

We analysed 321 primary cutaneous melanomas and 191 metastatic samples. In simple Cox regression, tumour thickness (p < 0.0001), presence of ulceration (p = 0.0001) and eTILs ≤16.6% (p = 0.0012) were found to be significant unfavourable prognostic factors for RFS. In multiple Cox regression, eTILs ≤16.6% (p = 0.0161) remained significant and downgraded the current staging. Lower eTILs in the primary tissue was associated with unfavourable relapse-free (p = 0.0014) and distant metastasis-free survival (p = 0.0056). In multiple Cox regression adjusted for tumour thickness and ulceration, eTILs as continuous remained significant (p = 0.019). When comparing TILs in primary tissue and corresponding metastasis of the same patient, eTILs in metastases was lower than in primary melanomas (p < 0.0001). In therapy-naïve metastases, an eTILs >12.2% was associated with longer progression-free survival (p = 0.037) and melanoma-specific survival (p = 0.0038) in patients treated with anti-PD-1-based immunotherapy. In multiple Cox regression, lactate dehydrogenase (p < 0.0001) and eTILs ≤12.2% (p = 0.0130) were significantly associated with unfavourable melanoma-specific survival.

**Interpretation:**

Assessment of TILs is prognostic in primary melanoma samples, and the eTILs complements staging. In therapy-naïve metastases, eTILs ≤12.2% is predictive of unfavourable survival outcomes in patients receiving anti-PD-1-based therapy.

**Funding:**

See a detailed list of funding bodies in the Acknowledgements section at the end of the manuscript.

**Research in context**

**Evidence before this study**

There are conflicting results regarding the prognostic significance of TILs in primary melanoma. *NN192* is an algorithm developed to standardise the method of TILs in primary melanoma. Moreover, no biomarkers are used in clinical practice to predict the outcomes of anti-PD-1-based immunotherapy.

**Added value of this study**

We validated that TILs quantification as eTILs using the deep-learning *NN192* algorithm is prognostic in primary melanoma in stages IB-IIC. We showed a decrease in eTILs from primary melanoma to matched metastases reinforcing the immunoediting hypothesis. We demonstrated that quantifying TILs in therapy-naïve melanoma metastases is predictive of response and survival outcomes in patients treated with anti-PD-1-based immunotherapy.

**Implications of all the available evidence**

eTILs complement staging for stage I/II melanoma patients to assess relapse-free survival, which could be applied in patients' follow-up and adjuvant therapy decisions. For early-stage BRAF-mutated melanoma, assessing whether patients should receive rather adjuvant BRAF inhibition due to low eTILs instead of immunotherapy is important. Currently, there are no predictive biomarkers in clinical practice for immunotherapy in stage III/IV melanoma patients. This study provides evidence that electronic quantification of TILs in melanoma metastases can be implemented as a predictive biomarker for immunotherapy. It highlights that patients with low eTILs are at higher risk of progression under immune checkpoint inhibition and that other options should be sought in these patients, i.e., BRAF/MEK inhibitors for BRAF-mutated melanoma, T cell therapies, and targeted therapy according to genomic alterations for BRAF wild-type melanoma.

## Introduction

Melanoma is an immunogenic tumour^1^ that often exhibits various cell types in its microenvironment, including fibroblasts, endothelial cells, and immune cells. The interaction of tumour cells with the microenvironment plays a critical role in melanoma progression, mediating either tumour immunity or tumour promotion. Of the immune cells, lymphocytes infiltrating the tumour are called tumour-infiltrating lymphocytes (TILs). Yazdi et al. showed that heterogenous T-cell clones could infiltrate primary melanomas.^2^ TILs include CD8+ T, CD4+ T, B cells, and NK cells, with CD8+ T cells being the most common subtype in melanoma and associated with a better prognosis. In contrast, other immune cells, including M2 macrophages, T-regulatory cells (Treg), and myeloid-derived suppressor cells (MDSC), act immunosuppressive, leading to tumour promotion.^3^^,^^4^ The distribution pattern of TILs in melanoma is heterogeneous, ranging from stromal to peritumoral and intratumoral. Intratumoral TILs are found within nests of melanoma cells, peritumoral TILs at the invasive margin of the tumour, and stromal TILs in the stromal areas beyond the tumour border.^5^^,^^6^

TILs have long been studied as a potential biomarker in melanoma, and various methods for assessing the immune infiltrate have been described. These include visual characterisation by a histopathologist, immunohistochemistry, multiplex immunofluorescence with quantification by image analysis tools, molecular methods based on gene expression signatures, determination of T-cell receptor (TCR)clonality, and proteomics.^7^ Different scoring and classification systems have been proposed for TIL evaluation and quantification. The most sophisticated system was proposed by Clark et al., in 1989, which characterises TILs as absent, non-brisk, and brisk.^8^ TILs are absent when no TILs are found within the tumour or exclusively in perivascular and fibrotic areas. Non-brisk infiltrate is defined as focal TILs found only at the tumour margins or the tumour base, whereas brisk TILs infiltrate the entire tumour or base.^9^^,^^10^ A recent meta-analysis showed that brisk lymphocytes were associated with better disease-specific survival.^11^ However, Němejcová et al. compared different scoring systems, and TILs were not found to be an independent prognostic factor when these were assessed histopathologically.^12^

The American Joint Committee on Cancer (AJCC) classification of melanoma (8th edition) is established for risk stratification of cutaneous melanoma patients based on overall survival. Tumour thickness, ulceration, and the presence of locoregional and distant metastases are the main criteria for stratifying patients. Early-stage cutaneous melanoma patients without metastases (stage I and II) are categorised into substages according to tumour thickness and ulceration.^13^, ^14^, ^15^ However, the relapse-free survival rate at 5 years of patients with a negative sentinel lymph node biopsy (SLNB) in Europe ranges from 76% to 90%, meaning that up to 25% of these patients relapse within 60 months despite the early stage of initial diagnosis.^16^^,^^17^ Additional and easy-to-determine biomarkers are needed to identify high-risk patients at early stages so that the follow-up strategies, such as close monitoring or adjuvant therapy, can be adapted. Accurate prognostic and predictive biomarkers could avoid overtreatment of patients with immune checkpoint inhibitors and prevent potential toxicity caused by these therapies.

Predictive biomarkers of response and survival are needed in clinical practice for advanced melanoma patients treated with anti-PD-1-based immunotherapy, as 40–60% of these patients do not benefit.^18^ Tumour mutation burden (TMB),^19^^,^^20^ circulating tumour DNA (ctDNA),^21^ specific genomic alterations,^22^ or gene expression scores are costly markers and are not available in every centre. Blood markers, such as lactate dehydrogenase (LDH) and neutrophil-to-lymphocyte ratio (NLR), are associated with increased tumour burden and elevated inflammation levels, respectively. They are generally linked to poor prognosis but have only a modest predictive value. Therefore, more reliable predictive biomarkers are needed to complement LDH, routinely assessed for staging. Tumeh et al. showed that responders to immunotherapy had higher numbers of CD8+, PD-1+, and PD-L1+ cells in treatment-naïve melanoma metastases than non-responders, raising the question of whether the quantification of TILs can be included in the staging.^23^

BRAF-mutated (BRAF^V600E/K^) melanomas account for 40% of cutaneous melanomas, and the presence of the BRAF^V600E/K^ is specifically predictive of response to the approved BRAF inhibitors. Immunotherapy, including anti-PD-1 antibodies, is effective in BRAF^V600E/K^ and BRAF-wild type (BRAF^wt^) melanoma. LDH is the only widely used predictive factor for both immune checkpoint inhibition and targeted therapy and is associated with poor prognosis for both therapies. More specific biomarkers are needed to predict resistance and response to immunotherapy, indicating which patients should receive immunotherapy and which should receive another treatment option.

In oncology, deep learning algorithms have recently revolutionised the field of biomarkers, leading to process standardisation. In many cancers, including colon,^24^ breast,^25^ and testicular cancer,^26^ the quantification of TILs was found to be associated with prognosis. In primary melanoma, earlier publications have evaluated the deep learning algorithms *NN192* and *ADTA* to assess TILs and demonstrated their prognostic significance independent of tumour thickness and ulceration. *ADTA* is based on the identification of patches, while *NN192* uses granular analysis.^27^, ^28^, ^29^ In metastatic melanoma, limited studies have addressed the prognostic and predictive significance of TILs quantification in hematoxylin-eosin-stained (H&E) sections.^10^^,^^30^^,^^31^

In our study, we investigated the prognostic significance of eTILs assessed in primary cutaneous melanoma tissue in terms of relapse-free survival (RFS), distant metastasis-free survival (DMFS), and overall survival (OS). Moreover, we assessed the association of TILs in metastatic tissue and survival outcomes, including progression-free survival (PFS) and melanoma-specific survival (MSS) after first-line anti-PD-1-based therapy and targeted therapy with BRAF and MEK inhibitors.

## Methods

### Study design

We included patients diagnosed with stage IB to IV cutaneous melanoma between 2010 and 2018. Patients were treated at the academic skin cancer centres in Tuebingen (Germany), Dresden (Germany), and St. Gallen (Switzerland). Other inclusion criteria were: (1) age ≥18 years, (2) availability of a H&E-stained slide of the tumour, (3) good quality of the H&E slide and (4) tumour percentage above >5%. Additional exclusion criteria were: (1) the presence of features not accurately detected by the algorithm, particularly necrosis and pigment incontinence, (2) patients with a tumour thickness of less than 1 mm as the algorithm did not accurately identify eTILs in these samples and are known to have a low risk for relapse, (3) brain metastases were excluded if the patients had received steroid treatment for the management of their brain metastasis prior to the acquisition of the sample (Supplementary Fig. S1a). Quality of the H&E slide was assessed by a dermatopathologist and aimed to include samples that were appropriately stained with hematoxylin and eosin, appropriately preserved/fixed and free of any artefacts or debris (blebs, folds, and bubbles) that may obscure or distort the tissue or cell morphology samples.

We assessed the primary tissue of stage I/II patients and the treatment-naïve metastatic sample of stage III/IV melanoma patients. 111 (34.6%) of patients diagnosed with stage I/II had a relapse (progression to stage III/IV) during the follow-up period (105 within 60 months). We also evaluated 89 H&E slides of the metastases excised between 2010 and 2019 that were available from these patients (Supplementary Fig. S1a and b).

### Ethics

The study was approved by the ethics committee at the medical faculty of the Eberhard-Karls-University Tuebingen (approval number 883/2019BO2), the Technical University of Dresden (EK 48022018), and by the ethics committee for Eastern Switzerland (EKOS 16/079) and was conducted following the Reporting Recommendations for Tumor Marker Prognostic Studies (REMARK) and the Transparent Reporting of a multivariable prediction model for Individual Prognosis Or Diagnosis (TRIPOD) guidelines.^32^^,^^33^ Individual consent was obtained for the use of patients' clinical data.

### Clinical data collection

The staging was performed according to the 8th Edition of the American Joint Committee on Cancer (AJCC) Staging Manual.^13^ A blinded investigator assessed the response to anti-PD-1-based immunotherapy using the Response Evaluation Criteria in Solid Tumors version 1.1 (RECIST 1.1).^34^ The Best Overall Response (BOR) was documented.

### Determination of the electronic TIL score (eTILs)

Based on our workflow, histopathological diagnosis of cutaneous melanoma was confirmed by a certified dermatopathologist using a H&E-stained slide (Supplementary Fig. S2) and slides with staining artefacts were excluded.

H&E-stained slides were digitised at a 40x magnification using a *Hamamatsu Nanozoomer* Digital Slide Scanner. The whole slide scans were analysed with the digital pathology software *Qupath* (version 0.1.2),^25^ and regions of interest (ROI) were marked under the supervision of a dermatopathologist. To refine the H&E stain estimates for each digitised slide and account for staining variation, we used the “estimate stain vectors” command in QuPath to set the stain and background vectors. Before running the command, we manually selected a small representative region containing clear examples of the staining and a representative background area. We employed watershed cell detection to segment the cells in the image using the following settings: detection image: hematoxylin OD; requested pixel size: 0.5 μm; background radius: 8 μm; median filter radius: 0 μm; sigma: 1.5 μm; minimum cell area: 10 μm^2^; maximum cell area: 400 μm^2^; threshold: 0.1; and maximum background intensity. The wand tool was used to select the tumour area and perform cell detection and segmentation. When this was not feasible in larger melanomas, we selected multiple regions or one large representative region from the invasive front up to the superior margin of the tumour. We performed visual segmentation quality control and added smoothed object features at 25 μm and 50 μm radius.

We used the network classifier *NN192,* which was trained to identify TILs compared to cancer cells, stromal and other cells on H&E sections in melanoma, as published by Acs et al.^27^ Using this algorithm that is publicly available on the *GitHub* platform, we calculated the eTIL score as eTILs = [TILs/(TILs + tumour cells)]∗100%^27^^,^^35^ (Supplementary Fig. S3). The evaluated TILs included intratumoral TILs within the predefined area and lymphocytes at the tumour base. Immune infiltrates beyond the invasive front were not calculated. The operator was trained on 30 melanoma samples and was blinded to the clinical data before using the algorithm.^27^ In 15 primary samples, the granular classifier for TILs identification in 15 primaries had an accuracy of 80%, F1 score of 82%, and recall of 79%, while in 15 metastases had an accuracy of 82%, F1 score of 79%, and recall 72%. Performance scores were assessed at the interface of tumour-infiltrating lymphocytes and tumour cells against visual classification and annotation of the cells in this region. All cases were checked for potential inaccuracies, such as misclassification of cells due to pigment incontinence or apoptotic cells, through visual inspection after neural network algorithm classification and excluded if necessary.

### Statistics

Summary statistics were reported. Categorical data were reported with counts (n) and percentages (%), and continuous variables were reported as a median and interquartile range [IQR]. We used the Wilcoxon-Mann-Whitney U test to compare continuous and categorical variables between two groups. For comparisons of a variable with more than two groups, the Kruskal–Wallis test was applied with Dunn's test for multiple comparisons. For comparisons between two categorical variables, Pearson's chi-squared test was used.

We calculated relapse-free survival (RFS), distant metastasis-free survival (DMFS), and overall survival (OS) of stage I/II patients as the time between the date of primary melanoma diagnosis and date of the event or the censoring date (last contact with the patient). Death due to all causes was considered an OS event. We dichotomised the stage I/II cohort using the published cut-off for eTILs of 16.6%.^27^^,^^29^

We assessed both scores for patients for whom both primary and metastatic tissue samples were available. A comparison between eTILs in primary tumours and their matched metastases was made using the non-parametric Wilcoxon matched-pairs signed-rank test and considering the type of metastatic tissue.

For stage III/IV, we assessed whether there is an association between TILs and survival outcomes in patients receiving PD-1 checkpoint inhibition or targeted therapy with BRAF and MEK inhibitors. The endpoints for this analysis were progression-free survival (PFS) and melanoma-specific survival (MSS). We defined PFS as the time between the start of systemic treatment and the date of progression or the last follow-up as the censoring date when no progression occurred. MSS was the time between the therapy start and the date of death or the last follow-up as the censoring date. The optimal cut-off that divided patients into two groups in terms of PFS was determined using the R package *“Evaluate Cutpoints*”.^36^

Censored data were analysed using the Kaplan–Meier method. A comparison of survival between the group with high eTILs (>16.6%) and low eTILs (≤16.6%) was made using the two-sided log-rank test. Median time-to-event and landscape rates were calculated using the Kaplan–Meier estimator, and hazard ratios (HR) were determined using the Cox Proportional Hazards Model. We used the likelihood ratio test to compare different nested models (stage versus stage plus eTILs group), and the Harrell concordance index (C-index, ranging from 0.5 and no concordance to 1.0 and perfect concordance of the model) was applied to compare the discriminatory ability of the models. The likelihood ratio test is used to compare two nested models, with the complex model differing only by the addition of some variables to the simpler model, and assesses whether this addition is statistically significantly different, making the complex model more accurate. Patient subgroups were defined according to their clinicopathological characteristics, and subgroup analysis was performed without correction for multiple comparisons.

We evaluated the effect of eTILs on staging by plotting Kaplan–Meier curves for patients stratified by both factors.

We conducted an evaluation of the linear relationship between the log-hazard (β coefficient) and the continuous eTILs. To test log-linearity, we plotted the continuous variable (eTILs) against the martingale residuals of a null Cox proportional hazard model. As this assumption was met, we proceeded to investigate the association between continuous eTILs and RFS and/or DMFS using the Cox proportional hazards model. To determine whether the linear model was appropriate, we used the likelihood ratio test to compare the model with linear eTILs to another model with restricted cubic splines of eTILs.

The concordance index (C-index) and likelihood ratio test were used to compare the model that included eTILs score and staging with the model based on staging alone. Furthermore, we plotted 5-year RFS against continuous eTILs for the different stages. Using the “regplot” package in R, we established a nomogram that incorporates eTILs score and staging to predict metastasis based on the results of the multiple Cox model.

For all analyses, two-sided tests were used, and p < 0.05 was considered statistically significant. Statistical analyses were performed using GraphPad Prism v.9.1.2 (GraphPad Software Inc., CA, USA) and R v.4.1.2 (R Core Team, Vienna, Austria) with “survival”, “survminer”, “forester”, “dynpred”, “Evaluate Cutpoints”, “regplot” packages.

### Role of the funding source

The funding sources were not involved in the study design, analysis, data interpretation, writing and submission of the manuscript.

## Results

### Early-stage melanoma cohort

#### Patient characteristics

We included 321 stage IB-IIC cutaneous melanoma patients. The primary tumour was excised between 2010 and 2018. At the time of diagnosis, 134 (41.7%) patients were in stage IB, 88 (27.4%) in stage IIA, 59 (18.4%) in stage IIB, and 40 (12.5%) in stage IIC. The median age of the patients was 67 years [IQR, 56–76], and the median follow-up in the cohort was 58 months [IQR, 37–88]. 57.3% of the patients were male. Most primary melanomas were non-ulcerated (n = 217, 67.7%) and non-regressive tumours (n = 187, 58.3%). The most common histological subtype was SSM (n = 156, 48.6%). Relapse occurred in 111 (34.6%) of patients during follow-up, with 94.6% (105 of 111) occurring within 5 years. An H&E-stained slide of metastatic tissue was available from 89 of these patients.

#### eTILs and clinicopathological features

The median eTILs, representing the frequency of tumour-infiltrating lymphocytes of all 321 primary tumours, was 22.10% [IQR, 14.77–32.19]. Of the primary tumours, 98 (30.5%) had eTILs below the published cut-off at 16.6%^27^ and 223 (69.5%) above 16.6% (Table 1). In terms of histological melanoma subtype, nodular melanomas (NM) showed lower eTILs compared to lentigo maligna melanoma (LMM) (p_adj_ = 0.0117, Kruskal–Wallis) and superficial spreading melanoma (SSM) (p_adj_ = 0.0021, Kruskal–Wallis). Regarding AJCC substages, stage IIC melanoma had the lowest eTILs, which were significantly lower than in stage IB (p_adj_ = 0.0008, Kruskal–Wallis) and IIA (p_adj_ < 0.0001, Kruskal–Wallis). Accordingly, melanomas thicker than 4 mm showed lower eTILs than tumours with a thickness of 1.1–2.0 mm (p_adj_ = 0.0002, Kruskal–Wallis) or 2.1–4.0 mm (p_adj_ = 0.0008, Kruskal–Wallis). The eTILs of primaries with regression were higher than those without regression (p = 0.0092, Mann–Whitney U) (Supplementary Table S1 and Supplementary Fig. S4a–d). There were no significant differences in eTILs between patients younger and older than 65 years, women and men, different locations of the tumour, BRAF^V600E/K^, and BRAF^wt^ melanomas, or between ulcerated and non-ulcerated melanomas (Supplementary Table S1 and Supplementary Fig. S5a–d).

**Table 1:** Baseline patient clinicopathological characteristics according to the eTILs grouped by the cut-off 16.6%.

Characteristics	eTILs ≤16.6%	eTILs >16.6%	p value
n = 321 (100%)	n = 98 (30.5%)	n = 223 (69.5%)	
**eTILs (%)**			**<0.0001**
Median [IQR]	11 [6.9–14.5]	28 [21.5–36.4]	
**Age at Dx (y)**			0.0775
Median [IQR]	68 [57–78]	66 [55–75]	
**Age group at Dx (y), n (%)**			0.1385
≤65	40 (26.5)	111 (73.5)	
>65	58 (34.1)	112 (65.9)	
**Sex, n (%)**			0.7735
Female	43 (31.4)	94 (68.6)	
Male	55 (29.9)	129 (70.1)	
**Localisation, n (%)**			0.1808
Head and neck	17 (26.6)	47 (73.4)	
Trunk	34 (28.8)	84 (71.2)	
Upper extr.	8 (21.6)	29 (78.4)	
Lower extr.	39 (38.2)	63 (61.8)	
**Histological subtype**^a^**, n (%)**			**0.0055**
SSM	42 (26.9)	114 (73.1)	
NM	31 (49.2)	32 (50.8)	
LMM	5 (19.2)	21 (80.8)	
ALM	17 (36.2)	30 (63.8)	
Unknown	3 (10.3)	26 (89.7)	
**BRAF V600 oncogenic variant**^a^**, n (%)**			0.2902
No	38 (25.7)	110 (74.3)	
Yes	21 (37.5)	35 (62.5)	
Unknown	39 (33.3)	78 (66.7)	
**Stage at Dx, n (%)**			0.3366
I	37 (27.6)	97 (72.4)	
II	61 (32.6)	126 (67.4)	
**Substage at Dx, n (%)**			**0.0058**
IA	NA	NA	
IB	37 (27.6)	97 (72.4)	
IIA	20 (22.7)	68 (77.3)	
IIB	20 (33.9)	39 (66.1)	
IIC	21 (52.5)	19 (47.5)	
**Tumour thickness (mm)**			**0.0036**
Median [IQR]	2.78 [1.55–5]	2 [1.4–3.22]	
**Tumour thickness group (mm), n (%)**			**0.0082**
≤1	NA	NA	
1.1–2.0	40 (25.2)	119 (74.8)	
2.1–4.0	30 (29.4)	72 (70.6)	
>4	28 (46.7)	32 (53.3)	
**Ulceration, n (%)**			0.1740
No	61 (28.1)	156 (71.9)	
Yes	37 (35.6)	67 (64.4)	
**Regression**^a^**, n (%)**			0.1051
No	64 (33.2)	129 (66.8)	
Yes	17 (23)	57 (77)	
Unknown	17 (31.5)	37 (68.5)	
**Survival (%)**			
5-year RFS	48.5	69.5	**0.0012**
5-year DMFS	68.2	86.8	**0.0007**
5-year OS	76.6	87.7	**0.0226**

Values are reported as counts (n) and percentages (%) for discrete values and as a median and interquartile range [IQR] for continuous values; for comparisons between categorical variables, Pearson's chi-squared test was used; for comparisons between a continuous and a categorical value, the Mann–Whitney U test was used; for comparisons of survival rates simple cox proportional hazards model was used; RFS, Relapse-free survival; DMFS, Distant metastasis-free survival; OS, Overall survival; SSM, Superficial spreading melanoma; NM, Nodular melanoma; LMM, Lentigo malignant melanoma; ALM, Acrolentiginous melanoma; y, Years; extr., Extremities; Dx, Diagnosis; CI, Confidence interval; NA, Not applicable.

a Patients for whom information was unknown were not used for comparisons; significant p values are in bold.

#### Survival analysis

Considering the median follow-up time of 58 months [IQR, 37–88] of our stage I/II cohort, we performed survival analysis at 5 years. The 5-year survival rates of the stage I/II cohort were 63.2% (95% CI, 57.2–68.6) for RFS, 81.3% (95% CI, 75.9–85.6) for DMFS, and 84.4% (95% CI, 79.4–88.3) for OS, respectively. Median RFS, DMFS, and OS were not reached. The 10-year follow-up data regarding RFS and DMFS showed the same trend with only few additional events (Supplementary Fig. S6).

**Fig. 1:**
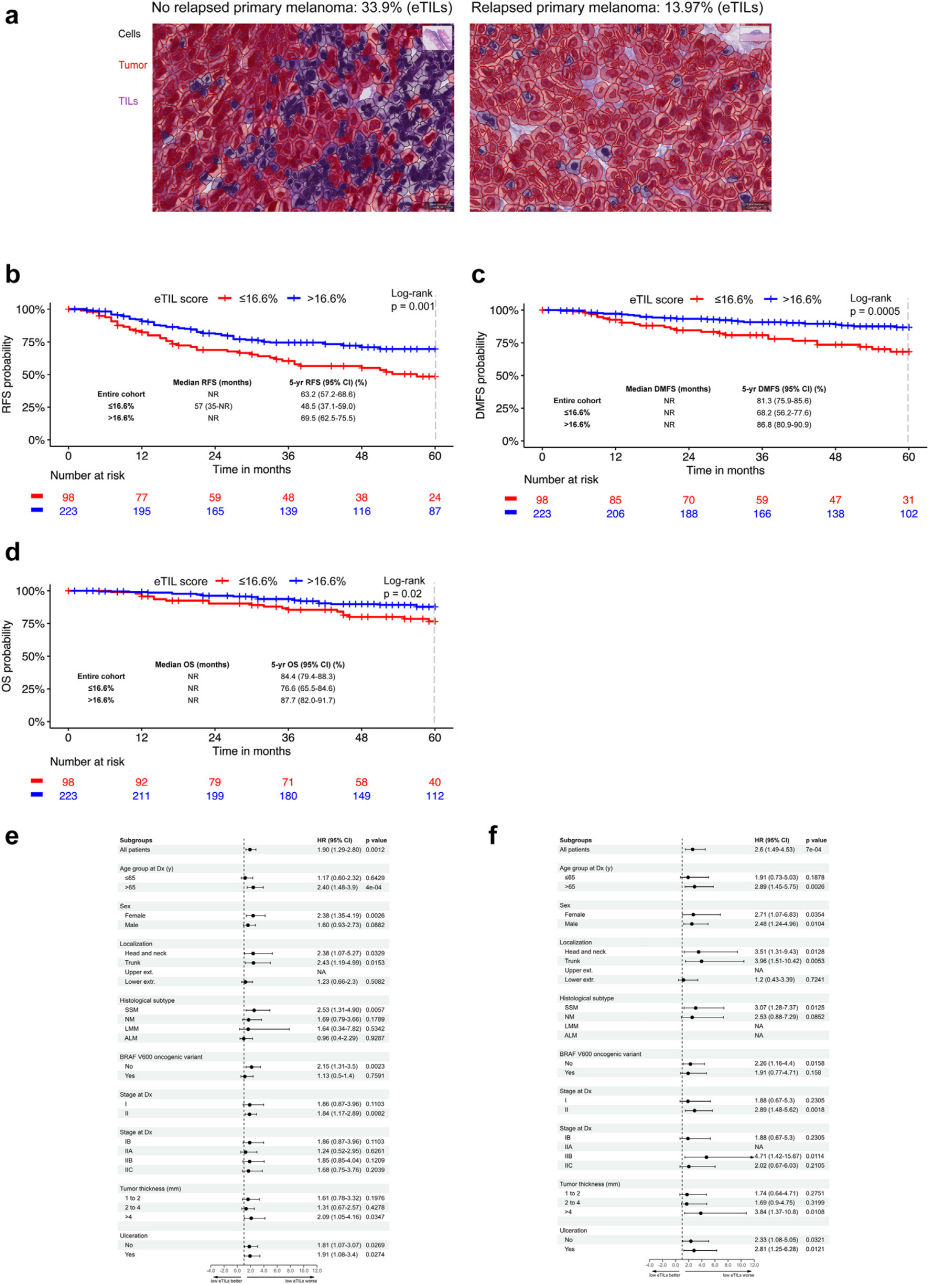
**eTILs is a prognostic biomarker in stage I/II cutaneous melanoma**. (a) Microphotograph showing a primary cutaneous melanoma with eTILs of 33.9% from a patient who did not develop a metastasis (left) and a melanoma with eTILs of 13.97% from a patient who did develop a metastasis (right). (b) Relapse-free survival analysis for the 321 stage IB to IIC patients according to the eTILs group using the cut-off of 16.6%. It shows the Kaplan–Meier curves for RFS and the number of melanoma patients at risk at specific time points starting from primary melanoma diagnosis. (c) Distant metastasis-free survival analysis for the 321 stage IB to IIC melanoma patients according to the eTILs group using the cut-off of 16.6%. It shows the Kaplan–Meier curves for DMFS and the number of melanoma patients at risk at specific time points beginning from the date of primary melanoma diagnosis. (d) Overall survival analysis for the 321 stage IB to IIC patients according to the eTILs group using the cut-off of 16.6%. It shows the Kaplan–Meier curves for OS and the number of melanoma patients at risk at specific time points starting from primary melanoma diagnosis. (e) Simple subgroup analysis for 5-year RFS for the low eTILs group ≤16.6% (reference: high group). The graph shows the unadjusted HR for RFS of the low eTILs group in the different patient subgroups. (f) Simple subgroup analysis for 5-year DMFS for the low eTILs group ≤16.6% (reference: high group). The graph shows the unadjusted HR for DMFS of the low eTILs group in the different patient subgroups. HR was not calculated if the events (relapses) were less than 10 in this subgroup. Abbreviations: HR, Hazard ratio; CI, Confidence interval; SSM, Superficial spreading melanoma; NM, Nodular melanoma; LMM, Lentigo malignant melanoma; ALM, Acral lentiginous melanoma; y, Years; extr., Extremities; Dx, Diagnosis; NA, Not applicable; HR was not calculated if fewer than ten events occurred in this patient subgroup; ∗Patients for whom information was unknown were not shown.

There was a statistically significant difference in RFS between the group with high (>16.6%) and low eTILs (≤16.6%) (p = 0.001, log-rank). The median RFS of the group with low eTILs was 57 months (95% CI, 35-NR), while it was not reached in the group with high eTILs. The 5-year RFS rate was 48.5% (95% CI, 37.1–59) in the low eTILs group and was inferior to this of the high eTILs group with 69.5% (95% CI, 62.5–75.5) (Fig. 1a and b).

DMFS was also significantly longer for the group with high eTILs (p = 0.0005, log-rank). The median DMFS was not reached for both groups. The 5-year DMFS was 68.2% (95% CI, 56.2–77.6) for the low eTILs group and 86.8% (95% CI, 80.9–90.9) for the high eTILs group (Fig. 1c).

With a hazard ratio of 2.6 (95% CI, 1.49–4.53; p = 0.0007), the risk of developing distant metastasis was more than double in the low eTILs group.

The overall survival (OS) curves of groups were also significantly different (p = 0.02, log-rank). Neither group reached the median OS. The 5-year OS rate was 76.6% (95% CI, 65.5–84.6) in the low group and 87.7% (95% CI, 82–91.7) in the high group (HR = 2.03; 95% CI, 1.10–3.72; p = 0.0226) (Fig. 1d).

In a subgroup analysis for 5-year RFS, the low eTILs group was associated with a significantly less favourable outcome for females, patients older than 65 years, patients with melanomas of the head, neck, and trunk, with SSM or BRAF^wt^ melanomas, with tumours thicker than 4 mm, or with ulcerated and non-ulcerated melanomas. Furthermore, in stage II patients, those with low eTILs had significantly worse RFS than those with high eTILs (Fig. 1e).

In a subgroup analysis of the 5-year DMFS, the low eTILs group performed significantly worse for both sexes, patients older than 65 years, patients with melanomas of the head, neck, and trunk, with SSM or BRAF^wt^ melanomas, with tumours thicker than 4 mm, or with ulcerated and non-ulcerated melanomas. Furthermore, in stage II patients, those with low eTILs had significantly worse DMFS than those with high eTILs (Fig. 1f).

In simple Cox regression for 5-year RFS, tumour thickness (p < 0.0001), ulceration (ref: no ulceration; HR = 2.1; 95% CI: 1.4–3.1; p = 0.0001) and eTILs (ref: high eTILs; HR = 1.9; 95% CI, 1.29–2.8; p = 0.0012) were significant unfavourable prognostic factors. Multiple cox regression for 5-year RFS adjusting for tumour thickness (p_adj_ = 0.0002) and ulceration (ref: no ulceration; HR = 1.44: 95% CI, 0.95–2.2; p_adj_ = 0.089) revealed eTILs ≤16.6% as an independent variable (ref: high eTILs; HR = 1.62; 95% CI, 1.09–2.41; p_adj_ = 0.0159) (Table 2).

**Table 2:** Simple and multiple Cox proportional hazards models for the 5-year RFS with eTILs as a categorical variable.

	Simple cox model				Multiple cox model			
	B	SE (B)	HR (95% CI)	p value	B	SE (B)	HR (95% CI)	p_adj_ value
Tumour thickness (mm)								
1.1–2.0	–			**<0.0001**	–			**0.0002**
2.1–4.0	0.83	0.24	2.3 (1.4–3.7)	**0.0004**	0.73	0.24	2.08 (1.3–3.4)	**0.0027**
>4.0	1.37	0.25	3.93 (2.4–6.3)	**<0.0001**	1.1	0.27	3 (1.8–5.1)	**<0.0001**
Ulceration								
No	–				–			
Yes	0.75	0.19	2.11 (1.4–3.1)	**0.0001**	0.37	0.22	1.44 (0.95–2.2)	0.0894
eTIL score (%)								
≤16.6	0.64	0.19	1.9 (1.3–2.8)	**0.0012**	0.48	0.2	1.62 (1.1–2.4)	**0.0159**
>16.6	–				–			

HR, Hazard ratio; CI, Confidence interval; SE, Standard error; p-values included in the model were calculated by the Wald test for each variable by dividing the coefficient by its standard error; statistically significant results are reported in bold.

To determine if eTILs adds discriminatory information to staging with respect to 5-year RFS, we calculated the C-index of the two corresponding models. The model estimating melanoma substages only (IB-IIC) had a C-index of 0.654 for RFS and 0.653 for DMFS, while the model, including eTILs, improved to 0.669 for RFS and 0.683 for DMFS, suggesting that the inclusion of the eTILs provided a better discriminative ability for both RFS and DMFS. We then compared the two nested models using the log-likelihood ratio test. The log-likelihood value of the model with the addition of eTILs was higher than the log-likelihood value with only the stage. This difference was statistically significant (p = 0.0173 for RFS, p = 0.0077 for DMFS), indicating that adding eTILs as a variable improves the goodness of fit and results in a more accurate model.

**Fig. 2:**
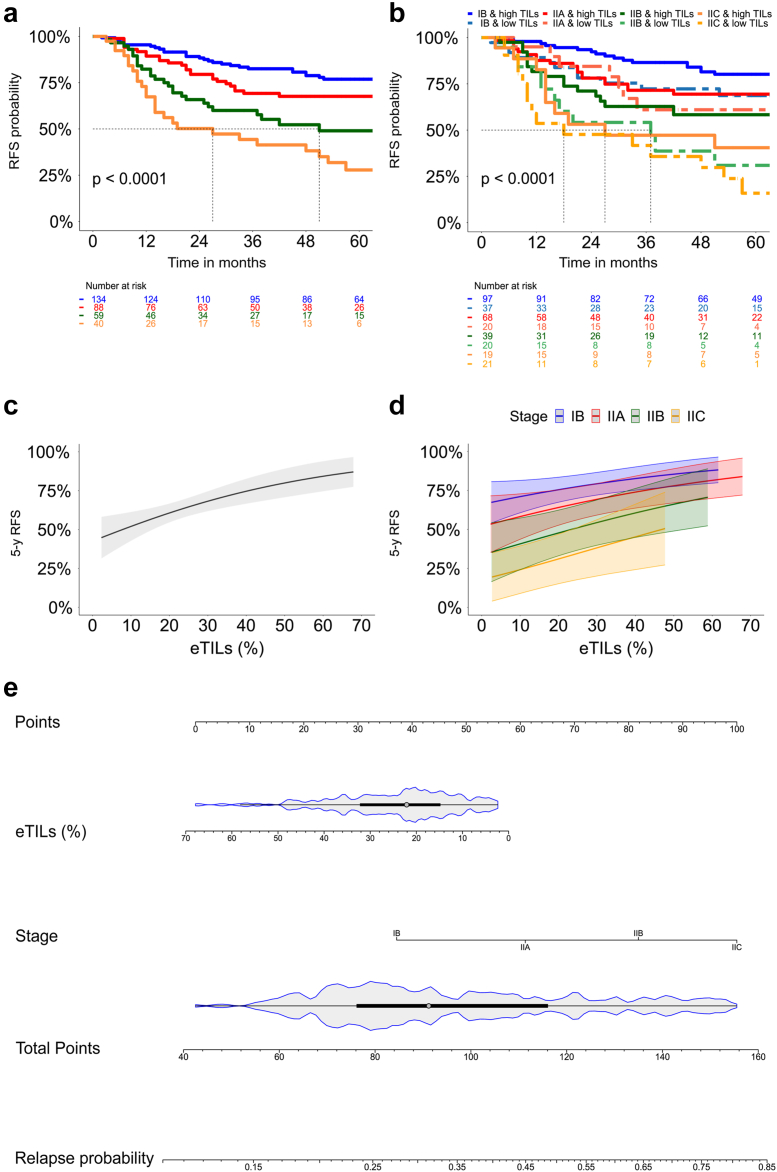
**The eTILs complements staging in stage I/II**. (a) Kaplan–Meier curve for relapse-free survival (RFS) according to the AJCC classification. (b) Kaplan–Meier curve for RFS according to the AJCC classification and the categorial eTILs (cut-off:16.6%). (c) Plot showing the relationship of continuous eTILs (%) and 5-year RFS probability. (d) Plot showing the relationship of the continuous eTILs (%) and the 5-year RFS probability for the different sub-stages. (e) Nomogram that predicts the probability of relapse within 60 months after melanoma diagnosis using the current staging and the continuous eTILs (%).

We used a Kaplan Meier plot for RFS to determine whether categorical eTILs up- or downgrade the current staging. The staging in our cohort showed 5-year RFS rates in accordance with the published ones (Fig. 2a). When we added the eTILs with the stated cut-off, low eTILs downgraded the staging (Fig. 2b).

Since, from a biological perspective, the more TILs, the better the prognosis, we also evaluated the prognostic significance of eTILs as a continuous variable. In the simple Cox regression, higher eTILs scores were associated with improved RFS (HR = 0.97; 95% CI, 0.96–0.99; p = 0.0015) and DMFS (HR = 0.97; 95% CI, 0.94–0.99; p = 0.0056). Furthermore, a near–linear relationship between 5-year RFS and continuous eTILs was found when we plotted both variables. This illustrates that higher eTILs scores correlate with a better prognosis in terms of 5-year RFS (Fig. 2c). The same relationship was observed when the data were divided by substages (Fig. 2d).

eTILs as a continuous variable remained significant (HR = 0.98, 95% CI, 0.97–0.99, p = 0.019) in multiple Cox regression for 5-year RFS, including tumour thickness (p = 0.0003) and ulceration (ref: no ulceration; HR = 1.45, 95% CI, 0.95–2.22, p = 0.0841) (Table 3). Both the staging (p < 0.001) and the eTILs (HR = 0.98, 95% CI; 0.96–0.99, p = 0.02) were significant in the multiple Cox regression for 5-year RFS. The C-index of the staging with eTILs as a continuous variable was 0.676 for RFS and 0.666 for DMFS. The likelihood ratio test revealed a significant superiority of the model that included eTILs (p = 0.0168 for RFS and 0.039 for DMFS). Continuous eTILs and staging were integrated into a nomogram to easily predict the relapse probability of early-stage melanoma patients (Fig. 2e).

**Table 3:** Simple and multiple Cox proportional hazards models for the 5-year RFS with eTILs as a continuous variable.

	Simple cox model				Multiple cox model				
	B	SE (B)	HR (95% CI)	p value	B		SE (B)	HR (95% CI)	p_adj_ value
Tumour thickness (mm)									
1.1–2.0	–			**<0.0001**	–				**0.0003**
2.1–4.0	0.83	0.24	2.3 (1.4–3.7)	**0.0004**	0.75		0.25	2.11 (1.3–3.4)	**0.0023**
>4.0	1.37	0.25	3.93 (2.4–6.3)	**<0.0001**	1.1		0.27	3.89 (1.7–4.95)	**0.0001**
Ulceration									
No	–				–				
Yes	0.75	0.19	2.11 (1.4–3.1)	**0.0001**	0.37	0.22		1.45 (0.95–2.2)	0.0841
eTIL score (%)^a^	−0.03	0.008	0.97 (0.96–0.99)	**0.0015**	−0.02	0.008		0.98 (0.97–0.99)	**0.0191**

a As continuous, HR, Hazard ratio; CI, Confidence interval; SE, Standard error; p-values included in the model were calculated by the Wald test for each variable by dividing the coefficient by its standard error; statistically significant results are reported in bold.

### Stage III-IV melanoma cohort

Next, we assessed 191 metastatic samples of stage III/IV cutaneous melanoma patients. The metastatic tumours were excised between 2010 and 2019. 89 metastases were matched to primary melanomas from patients in stage I/II cohort. Among the stage III/IV patients evaluated, 121 (63.4%) patients received anti-PD-1-based immunotherapy as first-line therapy, with 20 (16.5%) patients among them receiving adjuvant anti-PD-1. An additional 7 (3.6%) patients received ipilimumab, and 32 (16.8%) were treated with BRAF- or BRAF/MEK inhibitor-targeted therapies (Supplementary Table S2). A total of 30 (15.7%) metastatic patients did not receive any of these systemic treatments, as adjuvant therapy for R0 (microscopically margin-negative resection) patients had not yet been approved, patients received further treatment outside the clinic, received alternative therapies because of very rapid progression, or patients decided against system therapy. Metastasectomy or biopsy were conducted either as therapy (to obtain a R0 status) or for diagnostic purposes. eTILs of treatment-naive metastases was not significantly different between patients younger and older than 65 years (p = 0.8084, Mann–Whitney U), male and female patients (p = 0.6157, Mann–Whitney U), BRAF^V600E/K^ and BRAF^wt^ metastases (p = 0.2516, Mann–Whitney U), patients with presence or absence of brain metastasis at therapy start (p = 0.330, Mann–Whitney U) and patients with elevated or normal LDH at therapy start (p = 0.0913, Mann–Whitney U). However, there was a trend for patients with higher eTILs to have lower LDH at therapy start (median 15.02 vs 11.52). No significant differences were found. However, the median eTILs was highest in metastases of the lung (median:15.66 [IQR; 6.4–20.17]) and breast (median:17.85 [IQR; 9.9–33.2]), followed by in-transit (median:11.80 [IQR; 8.2–20.91]) and distant skin metastases (median:11.91 [IQR; 5.97–21.89]) and soft tissue (median:9.85 [IQR; 9.8–9.9]) or gastrointestinal metastases (median: 9.5 [IQR; 5.24–24.07]) (Supplementary Fig. S7).

#### Comparison of primary melanomas with matched metastases

**Fig. 3:**
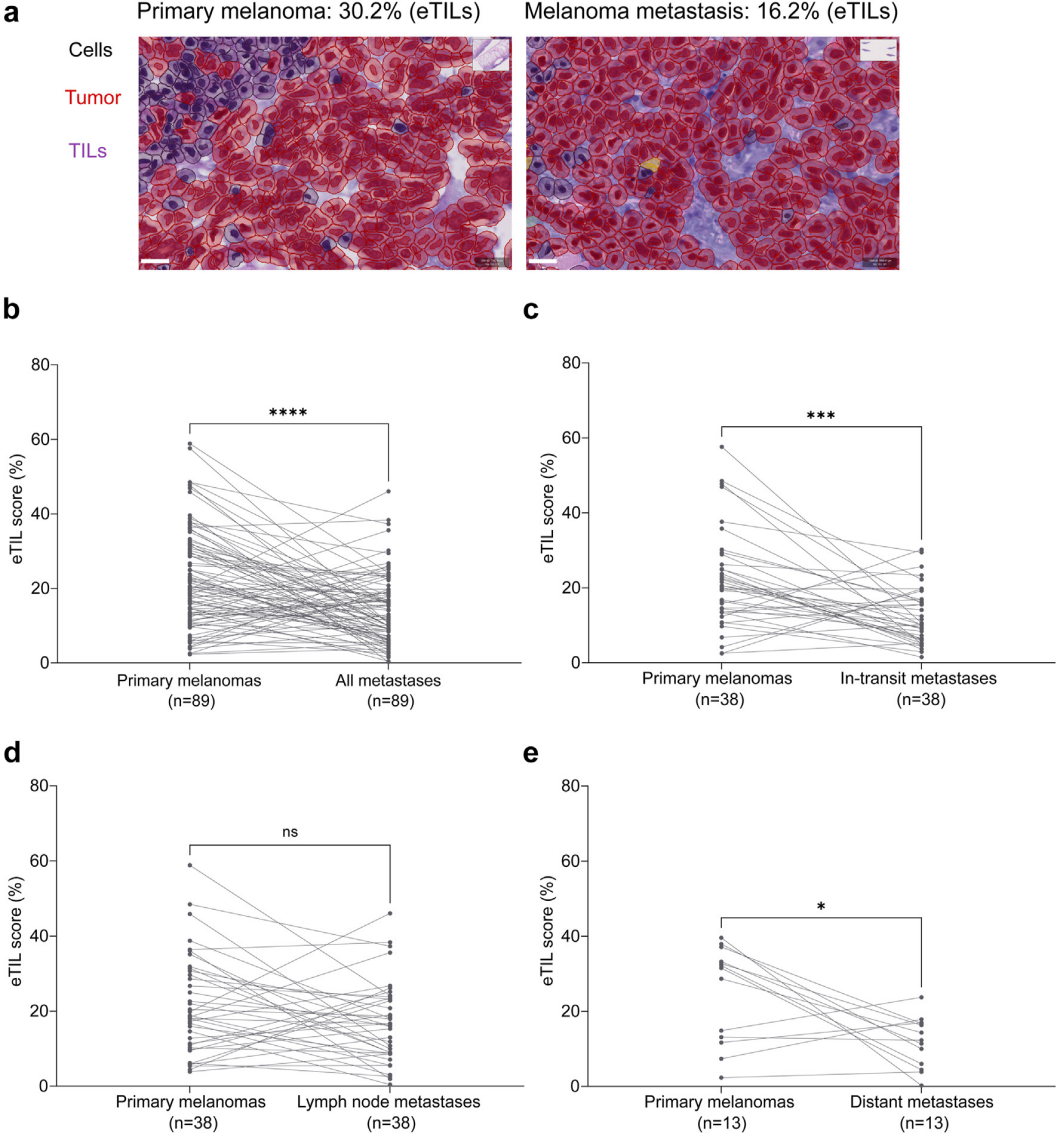
**Reduced TIL numbers in intraindividual melanoma metastases.** (a) Microphotograph showing a primary cutaneous melanoma with an eTIL score of 30.2% (left) and the matched metastasis from the same patient with an eTIL score of 16.2% (right). (b) Dot plot showing the eTILs of primary and metastatic tumours. Each line represents one patient. The Wilcoxon matched-pairs signed-rank test was statistically significant for a decrease in the eTILs from matched primary to metastatic samples (p < 0.0001). (c) Dot plot showing the eTILs of primary and matched in-transit metastases (Wilcoxon matched-pairs signed-rank test, p = 0.0002). (d) Dot plot showing the eTILs of primary and matched lymph node metastases (Wilcoxon matched-pairs signed-rank test, p = 0.0957). (e) Dot plot showing the eTILs of primary and matched distant metastases (Wilcoxon matched-pairs signed-rank test, p = 0.0266); ∗p < 0.05, ∗∗p < 0.01, ∗∗∗p < 0.001, ∗∗∗∗p < 0.0001.

We analysed 89 pairs of primary melanomas with matched intraindividual melanoma metastases. The median age at the time of relapse was 76 years [IQR, 67–83]. The evaluated metastatic samples included 38 (42.7%) in-transit metastases, 38 (42.7%) lymph node metastases (n = 28 regional and n = 10 distant), as well as 12 (15%) distant metastases (Supplementary Table S3). Of the metastatic specimens, 97% were obtained before starting the first-line systemic therapy (n = 86). The median eTILs of all metastases was 12.5% [IQR, 6.81–19.09]. Lymph node metastases, including regional and distant lymph node metastases, showed a median eTILs of 16.3% [IQR, 8.8–23.5], whereas the median eTILs of other metastases was 10.6% [IQR, 6.0–16.8]. Overall, the metastases had significantly lower eTILs compared to the corresponding primary melanomas (p < 0.0001, Wilcoxon matched-pairs signed-rank test) (Fig. 3a and b). The median reduction of the eTILs in metastases was −8.125% (95% CI, −11.18 to −4.786) compared to the matched primaries. This reduction was significant for in-transit and distant skin and visceral metastases, while the decrease in lymph node metastases was not statistically significant (Fig. 3c and d).

### Characteristics of patients receiving non-adjuvant anti-PD-1 therapy

**Fig. 4:**
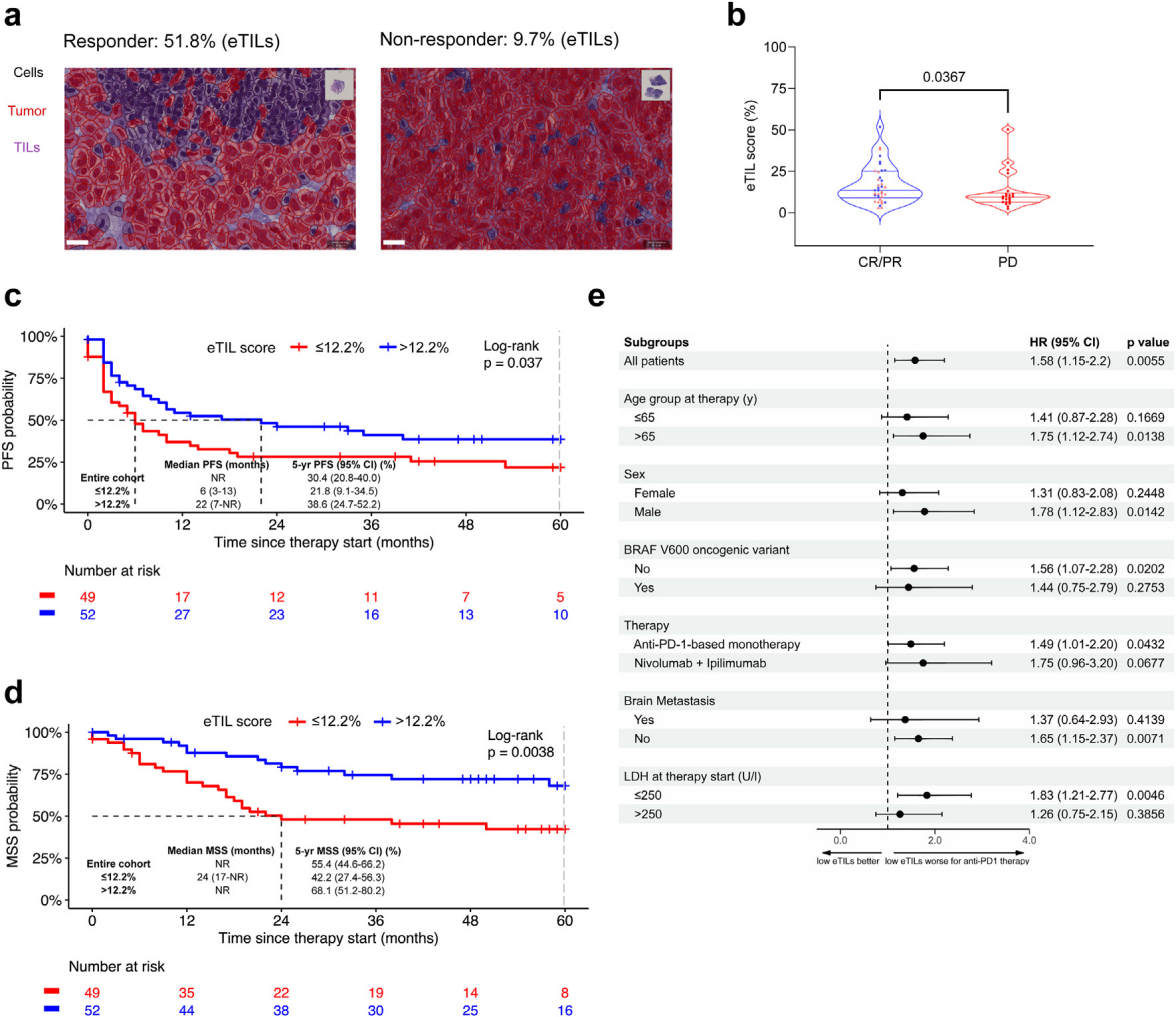
**eTILs is a predictive biomarker for non-adjuvant anti-PD-1 checkpoint inhibition in advanced melanoma patients.** (a) Microphotograph showing the evaluated metastasis from patient, who responded to immunotherapy and had an eTIL of 51.8% (left), and the metastasis from patient, who did not respond and had a score of 9.7% (right). (b) Violin plots of patients achieving an objective response (CR/PR) under anti-PD-1-based immunotherapy as compared to the non-responders (PD) (p = 0.0367, Mann–Whitney U). (c) PFS analysis for the 5-year follow-up after anti-PD-1-based therapy initiation for the 101 stage III to IV patients according to the eTILs group using the cut-off 12.2%. It shows the Kaplan–Meier curves for PFS and the number of melanoma patients at risk at specific time points. (d) MSS analysis for the 5-year follow-up after anti-PD-1-based therapy initiation for the 101 stage III to IV patients according to the eTILs group using the cut-off 12.2%. It shows the Kaplan–Meier curves for MSS and the number of melanoma patients at risk at specific time points. (e) Simple subgroup analysis for 5-year MSS for the low eTILs group ≤12.2% (reference: high group). The graph shows the unadjusted HR of the low eTILs group in the different patient subgroups of the patients treated with anti-PD-1-based therapy. Abbreviations: HR, Hazard Ratio; CI, Confidence Interval.

The median age of melanoma patients (n = 101) receiving non-adjuvant, first-line systemic anti-PD-1-based therapy was 68 years [59–77]; 63% were men, and 27% of the tumours had an oncogenic mutation in the *BRAF* gene. Of these patients, 41% received combined anti-PD-1 therapy (ipilimumab plus nivolumab), and 59% received pembrolizumab (40%) or nivolumab (19%) as monotherapy. Most of the patients (46%) were at stage M1c, followed by M1d (18%) and M1b (18%) (Table 4). Only 9 (8.9%) patients were in stage III. The median follow-up time after treatment initiation was 57 months (95% CI, 48–63). TILs were quantified as before (Fig. 4a), revealing a median eTILs of 13% (95% CI, 8–21). Moreover, patients achieving an objective response (CR/PR) had higher eTILs than the non-responders (PD) (p = 0.0367, Mann–Whitney U) (Fig. 4b).

**Table 4:** Patient and disease characteristics of those whose metastatic sample was assessed.

Characteristics	Non-adjuvant anti-PD-1-based therapy, n = 101
Matched with primary, n (%)	
Matched	31 (31)
Not matched	70 (69)
Centre, n (%)	
Dresden	4 (4)
St. Gallen	12 (12)
Tuebingen	85 (84)
eTILs (%), median [IQR]	13 [8–21]
Sex, n (%)	
Female	37 (37)
Male	64 (63)
Age at therapy (yrs), median [IQR]	68 [59–77]
Age at therapy (yrs), n (%)	
≤65	41 (40.6)
>65	60 (59.4)
Therapy, n (%)	
Anti-PD-based monotherapy	59 (59)
Nivolumab + Ipilimumab	42 (41)
Stage at therapy, n (%)	
IIIA	1 (1.0)
IIIB	1 (1.0)
IIIC	7 (6.9)
IIID	–
IV-M1a	10 (9.9)
IV-M1b	18 (18)
IV-M1c	46 (46)
IV-M1d	18 (18)
*BRAF* mutation, n (%)	
Mutated	27 (27)
Wt	72 (71)
Unknown	2 (2.0)
Brain Metastases, n (%)	
Metastases	18 (18)
No metastases	83 (82)
LDH at therapy start (U/l), median [IQR]	223 [184–300]
LDH at therapy start (U/l), n (%)	
≤250	20 (62.5)
>250	12 (37.5)

Values are reported as counts (n) and percentages (%) for discrete values and as a median and interquartile range [IQR] for continuous values.

### Survival analysis

We applied the *Evaluate Cutpoints* package in R^36^ using progression-free survival (PFS) as an endpoint to determine the optimal threshold for eTILs that separated the cutaneous advanced unresectable melanoma patients that received anti-PD-1-based immunotherapy (n = 101) into two prognostically different groups. We obtained 12.2% as the optimum cutpoint for the eTILs, and those with ≤12.2% (low eTILs) had a significantly shorter PFS (p = 0.037, log-rank) and melanoma-specific survival (MSS) (p = 0.0038, log-rank) than those with eTILs >12.2% (high eTILs). The median PFS in the low eTILs group was only 6 months (95% CI, 3–13), while it was 22 months (95% CI, 7-NA) in the high eTILs group (Fig. 4c). The median MSS in the low eTILs group was 24 months (95% CI, 17-NA), while it was not reached in the high eTILs group. Accordingly, the 5-year MSS was 68.1% (95% CI, 51.2–80.2) in the high eTILs group compared to 42.2% (95% CI, 27.4–56.3) in the low eTILs group (Fig. 4d).

In simple Cox regression, eTILs≤12.2% (HR = 1.58; 95% CI, 1.15–2.20; p = 0.0055), sex (HR = 1.39; 95% CI, 1.02–1.89; p = 0.0381) and LDH as a continuous variable (p < 0.0001) were significantly associated with MSS.

LDH (p < 0.0001) and eTILs≤12.2% (p = 0.0130) remained significantly unfavourable variables in a multiple Cox regression of MSS data (Table 5).

**Table 5:** Simple and multiple Cox proportional hazards models for 5-year MSS in 101 advanced melanoma patients treated with first-line anti-PD-1-based immunotherapy.

	Simple cox model		Multiple cox model	
	HR (95% CI)	p value	HR (95% CI)	p value
eTILs(%)				
≤12.2	1.58 (1.15–2.20)	**0.0055**	1.52 (1.09–2.11)	**0.0130**
>12.2	–		–	
**Age at therapy (yrs)**				
≤65	1.08 (0.57–2.01)	0.8211	NA	
>65	–			
**Age at therapy (yrs)**	1.00 (0.97–1.02)	0.8426	NA	
**Sex**				
Male	–		–	
Female	1.39 (1.02–1.89)	**0.0381**	1.22 (0.88–1.68)	0.2279
**Type of anti-PD-1 therapy**				
Monotherapy	1.55 (0.79–3.05)	0.2047	NA	
Combination	–			
***BRAF* mutation**				
Wt	1.40 (0.67–2.94)	0.3780	NA	
Mutant	–			
**Brain Metastases**				
Yes	1.12 (0.75–1.69)	0.5726	NA	
No	–			
**LDH at therapy start: U/l**				
≤250	–			
>250	1.23 (0.65–2.32)	0.5195	NA	
**LDH at therapy start; U/l**	1.01 (1.01–1.01)	**<0.0001**	1.01 (1.01–1.01)	**<0.0001**

The subgroup analysis of 5-year MSS showed the same trend toward worse prognosis for patients being categorised as low eTILs (≤12.2%) for all subgroups (Fig. 4e). In terms of the type of immunotherapy, low eTILs was an unfavourable predictive factor for both patients receiving anti-PD-1 monotherapy (HR = 1.49; 95% CI, 1.01–2.2; p = 0.0432) and combination immunotherapy (nivolumab + ipilimumab) (HR = 1.75; 95% CI, 0.96–3.2; p = 0.0677).

In patients receiving targeted therapy (n = 32), there was no significant difference in prognosis according to the eTIL score (PFS: p = 0.31; MSS: p = 0.44, log-rank). In patients receiving adjuvant anti-PD-1-based therapy (n = 21), there was a trend toward shorter PFS for the group of patients with low eTILs (≤12.2%), but neither PFS (p = 0.24, log-rank) nor MSS (p = 0.72, log-rank) reached significance due to the small sample size (Supplementary Fig. S8a–d).

## Discussion

Our study is a large-scale study that first assessed the prognostic relevance of eTILs in patients diagnosed at early stages. Primary tissue of 321 early-stage melanoma patients with a 5–10-year follow-up period was assessed, and therefore robust RFS, DMFS, and OS data were analysed. RFS and DMFS were considered optimal endpoints. The primaries of stage IB and IIC were excised before 2018, before the approval of early-stage adjuvant therapies, and therefore RFS and DMFS were not influenced by potential systemic therapy. We demonstrated the prognostic relevance of eTILs as a continuous variable in primary early-stage (IB-IIC) cutaneous melanoma, in addition to tumour thickness and ulceration as the main determinants of disease stage. We validated the published eTILs cut-off of 16.6%^27^ by finding that the two groups with high and low eTILs had significantly different RFS, DMFS, and OS, with the low eTILs group having the worse prognosis. The eTILs remained significant in the multiple Cox regression analysis adjusted for tumour thickness and ulceration; variables currently included in the actual AJCC staging (8th edition). This result is consistent with previous publications associating eTILs with disease-specific and relapse-free survival in melanoma patients.^27^^,^^29^^,^^37^

The conflicting results of previous publications regarding the prognostic value of TILs in primary melanoma assessed by histopathologists were most likely due to a lack of standardisation of the quantification method.^12^ With the introduction of *NN192*, there was the first evidence for the general prognostic significance of TILs quantified with the help of machine learning in cutaneous melanoma.^37^ These results render eTILs calculated with the deep learning algorithm *NN192* a robust prognostic biomarker. eTILs could complement AJCC staging for stages IB through IIC to assess the risk of relapse and metastasis, as indicated in our analysis by the increase in the C-index after adding eTILs to the staging and by the log-likelihood ratio test showing that the two nested models were significantly different. As adjuvant therapies are approved for earlier stages, there is a need for a reliable and easy-to-perform risk assessment for RFS and DMFS. Consequently, patients having low eTILs are at higher risk of relapse and distant metastasis and could be offered closer follow-up or appropriate adjuvant therapies.^27^^,^^28^ The prognostic value was independent of the BRAF mutation status, as shown by the subgroup analysis of patients with either BRAF wild type or BRAF mutated melanoma.

Our observed reduction in TILs from primary melanoma to matched metastasis supports the immunoediting hypothesis that immune escape and antigen modification during the multistep process of metastasis lead to reduced infiltration of tumour tissue by immune cells that suppress tumour growth. This observation is consistent with findings in breast cancer, showing that the tumour microenvironment influences progression and immune evasion and therefore is crucial for the development of metastases.^38^, ^39^, ^40^ We showed that the in-transit and distant metastases had significantly lower eTILs than the matched primary melanomas, although this decrease was not significant for lymph node metastases. One explanation for this would be that the algorithm cannot discriminate normal lymphocytes in lymph nodes and TILs.

As a further point, we have assessed the predictive relevance of eTILs in advanced stages, where systemic therapy is needed. By analysing 101 treatment-naïve metastatic samples of patients receiving non-adjuvant first-line anti-PD-1-based ICI after entering stage III/IV, we demonstrated that patients with high eTILs in their metastasis had better PFS and MSS than those with lower scores. Most metastases were skin metastases, either satellites/in-transit or distant skin metastases. We focused on patients treated with first-line immunotherapy, as previous therapies could alter the tumour microenvironment, leading to increased infiltration of the tumour by cells with immunosuppressive capabilities. Tumeh et al. already showed that responders to immunotherapy had more CD8+, PD-1+, and PD-L1+ cells in their samples than non-responders.^23^ Our data extend this result to survival outcomes and propose using the eTILs in the clinic, which measures lymphocytes, including CD8+ cells and other subtypes,^37^ as a predictive factor for response to anti-PD-1-based immunotherapy and survival of advanced cutaneous melanoma patients. Similar results were demonstrated in other cancers, showing that machine learning-based calculations of TILs were associated with response to immunotherapy.^41^ eTILs ≤12.2% and LDH remained significant in multiple Cox regression, making the combination a good predictor of survival outcomes after immune checkpoint inhibition. The eTILs appears to be a more specific biomarker for immune checkpoint inhibition than LDH, which could help identify patients who should or should not receive ICI. For targeted therapies with BRAF/MEK inhibitors, there was no statistically significant difference between low (≤12.2%) and high eTILs (>12.2%). However, we cannot make a general conclusion due to the small sample size. Recently a publication showed that TILs were predictive of cutaneous immune-related adverse events in advanced melanoma patients treated with PD-1 inhibitors.^42^ Considering that the presence of cutaneous toxicities has been associated with better response outcomes, this reinforces our findings.^43^ Moreover, a phase I trial showed that studies assessing CD8+ TILs in cancer lesions using PET-CT is safe and can be predictive of response outcomes.^44^

CD8+ cells, M0, and M2 macrophages represent melanoma's most frequent immune cell populations.^45^ Therefore, the eTILs calculated by *NN192* are mainly determined by CD8+ cells and macrophages.^45^ Antoranz et al. showed that the spatial distribution of cytotoxic T cells and PD-L1+ macrophages predicted response to anti-PD-1 immunotherapy in melanoma.^46^ Using in-depth single-cell RNA data, Tirosh et al. revealed the variability in the activation states and the clonal expansion of T cells across melanoma patients.^47^ Therefore, the location and functional characteristics of TILs within the melanoma microenvironment can have a different prognostic and predictive impact. Immune cells, according to their exact subtype and their activation status, whether they are exhausted, effector T cells or effector memory cells, have a different impact on tumour progression (for example, Th1, Th2, Th9, Th17, Th22, or Treg for CD4+ T cells; M0, M1, M2 for macrophages; Tc1, Tc2, Tc9, Tc17, Tc22 for CD8+ T cells; cDC1, cDC2, pDc, moDc, iDc, Langerhans cells for dendritic cells; or N1 and N2 for neutrophils). Through cytokine secretion, the CD8+ and CD4+ Th1 T cells are the primary effector cells associated with a good prognosis, while other CD4+ T cell subsets (Th2, Th17), myeloid suppressor cells, M2 macrophages, and Treg cells cause tumour promotion.^48^ Also, high pre-treatment clonality of the TILs has been associated with better OS of melanoma patients treated with anti-PD1 therapies.^49^

As a consequence of our results, patients with high eTILs and a relevant risk of relapse (IIB and above) or advanced melanoma patients (stage III/IV) should receive anti-PD-1 therapy for both BRAF wild type and BRAF-mutated melanoma. This concept is also supported by the DREAMseq trial demonstrating that patients with BRAF-mutated melanoma receiving first-line ICI and then targeted therapy had a better prognosis than those receiving initially targeted therapy and, subsequently, ICI. However, TILs or CD8+T cells were not assessed in this trial.^50^ Klein et al. also showed that clusters of TILs in BRAF^V600E/K^ melanoma were more likely to be associated with a better prognosis and response to immunotherapy than in NRAS-mutated or BRAF/NRAS wild-type melanoma.^31^

Concerning BRAF-mutated melanoma with low TILs, the optimal therapeutic strategy should be sought, as BRAF inhibition can have immunomodulatory effects by increasing the number of TILs or reprogramming them.^51^ Ascierto and colleagues demonstrated that patients (stage IIC to IIIC) with BRAF^V600E/K^ melanoma and low TILs had a greater benefit in terms of disease-free survival after adjuvant BRAF inhibition (vemurafenib) compared to those with high TILs.^52^ Therefore, targeted therapy with BRAF inhibitors may be an option for the group with low eTILs either as a combination or as a sequential therapy. Clinical trials evaluating TILs should address this topic in the future.

Since patients with low eTILs seem to benefit less from anti-PD-1-based therapy. Novel therapies such as adoptive T-cell therapy, T-cell engagers, or novel targeted therapy strategies according to the oncogenic driver alterations might be alternatives for BRAF^wt^ melanoma patients. Phase I and II have shown that autologous TILs therapy is a promising alternative.^53^ Integrating the use of multiple imaging modalities and genetic data should be done to optimise patient selection. Regarding adjuvant therapy in patients with low TILs, clinical trials are needed to determine the number of patients required for treatment and the number required for damage to determine which patients should be treated with what type of therapy, which may be more effective than anti-PD1 immune checkpoint blockade in this collective.

How clinicians can apply *NN192* or other deep-learning algorithms to cutaneous melanoma patients remains to be addressed. The guideline-compliant treatment for stage I/II melanoma is the complete surgical removal of the primary melanoma and adjuvant immunotherapy for stages IIB/IIC.^54^ Our results show that low eTILs downgrades staging throughout the early stages. Therefore, patients in stages IB to IIA with high TILs should be followed up according to guidelines. In contrast, patients with low eTILs should be monitored more closely or even considered candidates for adjuvant therapy from our point of view. We propose a model of how TIL can be applied in the clinic regarding follow-up and therapeutic strategies (Supplementary Fig. S9). Clinical trials are needed to investigate this.

In several other cancers, including breast, lung, and colorectal cancer, TILs are accepted as prognostic and predictive biomarkers. In breast cancer, TILs are considered a marker of tumour immunogenicity. Higher levels of stromal TILs were strongly associated with better prognosis in early-stage triple-negative breast cancer and HER2-positive breast cancer and better clinical outcomes in patients treated with adjuvant, neoadjuvant chemotherapy, or PD-1 inhibitors. The International Immuno-Oncology Biomarker Working Group, also known as the International TILs Working Group, has recently developed guidelines for evaluating TILs in breast cancer, proposing a standardised scoring system that considers the percentage and distribution of TILs and has recommended its use in clinical trials.^5^^,^^45^^,^^48^^,^^55^, ^56^, ^57^ With the help of this, TILs were shown to be predictive for therapy with the anti-PD-1 pembrolizumab but not for chemotherapy using anthracyclines or/and taxanes in a clinical trial for metastatic triple-negative breast cancer.^58^ CD8-positive TILs are also associated with better prognosis and response to therapy in non-small cell lung cancer.^41^^,^^59^^,^^60^ Similarly, TILs were found to be beneficial in colorectal cancer, leading to the well-known example Immunoscore, which measures tumour CD3+ and CD8+ lymphocyte density and has been incorporated into the current staging system (TNM-I).^24^ TILs are also associated with prognosis in several other types of cancer, including head and neck, gastric and ovarian cancer.^61^, ^62^, ^63^

Along with the advantages of the present study, some limitations remain. First, it is a retrospective study. The scanner used to digitise the H&E stains differed from the one used to develop the algorithm. Although it is an automatic algorithm, the process can only be considered semi-automatic because it is operator-dependent, and intensive operator training was essential for the accurate quantification of TILs and melanoma cells. A misclassification of cells was initially avoided by calculating the performance metrics and obligatory visual inspection, but the possibility of an error stills remains for the granular classifier. Immune cells beyond the invasive tumour margin, immune cell subtypes, phenotypes, spatial localisation, or the functional status of TILs were not analysed, and we did not evaluate tertiary lymphoid structures or germinal centres.^29^ Most metastases assessed were distant skin or in transit and satellites. A few high-score patients developed metastasis in the stage I/II cohort, which may be attributed to lymphocytes with tumour-suppressive capacities or cells misclassified as TILs. A more detailed characterisation of these samples in the future will provide information on how to improve the accuracy of the eTILs or whether additional staining is required for certain markers. Although we observed a difference in progression-free survival (PFS) using the eTILs cut-off in metastatic samples at baseline, several patients with high scores exhibited disease progression, highlighting the need for a combination of biomarkers that can better discriminate these patients.^18^, ^19^, ^20^^,^^64^, ^65^, ^66^, ^67^ Such additional biomarkers could be tumour mutational burden, specific mutations, microsatellite instability, microenvironmental factors and many others.

Further research is needed, and challenges exist before implementing such deep-learning scores in the clinic.^68^ The inclusion of eTILs or similar TIL scores, which could even be generated by manual counting if slide scanners are not available, in the pathology report is the first step that makes extensive multicentre studies possible. They should assess the variability among operators, slide scanners, and centres due to different pathology processing methods. However, standardisation is essential. The International TILs Working Group has already developed guidelines for scoring TILs in breast cancer, widely used in clinical and research settings. The development of guidelines for H&E-based TILs assessment in melanoma, similar to those developed for breast cancer, should be performed. They should include recommendations for the staining protocol, the definition of the region of interest and the quantification method. It should also be addressed how to simplify the deep learning classifiers in a user-friendly manner and how to standardise their use.^28^ While machine learning algorithms can increase the efficiency and accuracy of TIL classification and quantification, a trained pathologist must review and verify the results of these algorithms, and a training phase is essential prior to implementation to adapt the classifier to the centre's specifications to achieve optimal accuracy in TIL enumeration. Acknowledging that deep learning classifiers are not a substitute for human expertise is important. Therefore, pathologists should be trained appropriately. They should learn how to fine-tune the algorithms, evaluate their performance and be aware of potential limitations and pitfalls.

**Fig. 5:**
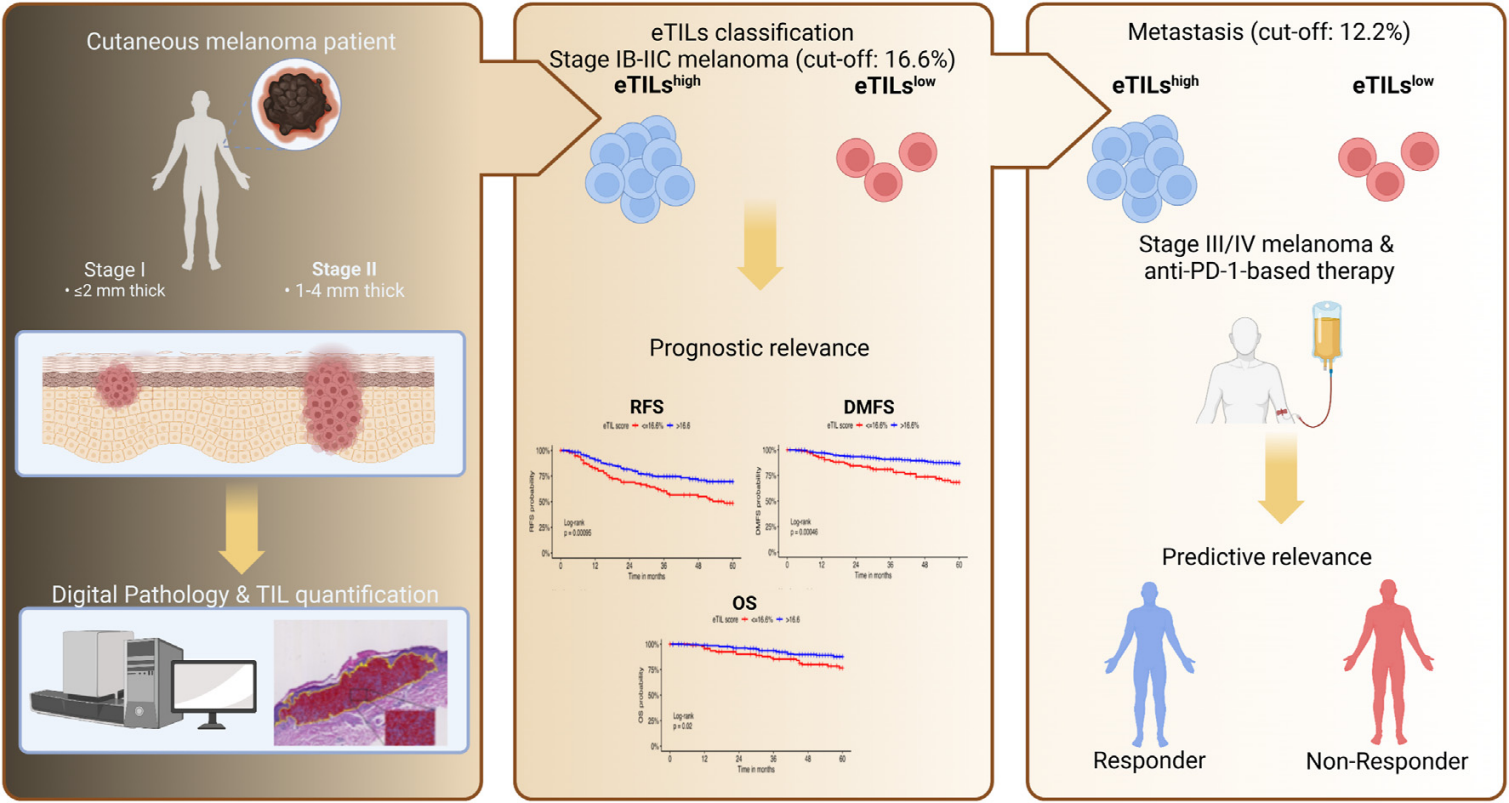
**Graphical summary of the prognostic and predictive relevance of eTILs in cutaneous melanoma**. eTILs could be used as a prognostic biomarker in primary melanoma that complements staging and as a predictive biomarker in melanoma metastases for anti-PD-1-based immunotherapy.

Overall, the enumeration of TILs as eTILs is an affordable and technically feasible prognostic and predictive biomarker based on simple diagnostic H&E staining and a publicly available algorithm without the need for costly methods, including sequencing or immunohistochemistry (Fig. 5).

The present study confirms the prognostic significance of TILs in early-stage primary cutaneous melanoma and shows that they could complement the current AJCC classification for determining the risk of relapse. Our results also show that eTILs in therapy-naïve cutaneous melanoma metastases are a potential predictive biomarker for response and survival outcomes to anti-PD-1-based immunotherapy.

## Contributors

Conceptualisation: DLR, HN, TA, TS; methodology: DLR, TNA, EC, JR, TA, TS; data curation: EC, SF, JR, UK, HN, LKC, DW, JS, OTP, TA, TS; verification of the underlying data: EC, JR, TA and TS; formal analysis: EC, JR, TA, TS; investigation: EC, JR, TA, TS; resources: SF, LF, TE, CG, FM, IB, DLR, MR; visualisation: EC, TS; supervision: TE, LF, MR, TA, TS; project administration: HN, TA, TS; funding acquisition: HN, TA, TS; writing—original draft: all authors; writing—review & editing: all authors.

## Data sharing statement

All scanner-based imaging data is centrally managed through the University-wide data management infrastructure at the Quantitative Biology Center (QBiC) using the Open Microscopy Environment (OME) Remote Objects (OMERO) infrastructure.^69^ The imaging metadata is standardised using the bio-formats framework.^70^ All data is accessible via qbic. life and can be made available upon reasonable request and with permission of the corresponding author after an appropriate data access agreement specifying the terms and conditions of use of the data.

## Declaration of interests

SF received personal fees from Kyowa Kirin and Takeda Pharmaceuticals, institutional grants from NeraCare, SkylineDx, and BioNTech, all outside the submitted work.

TA reports institutional grants and personal fees from Novartis, institutional grants from NeraCare, Sanofi, SkylineDx, personal fees from CeCaVa, Pierre Fabre, BMS all outside the submitted work, participate on a data safety monitoring board for Unicancer.

DLR reports grants and personal fees from Amgen, Astra Zeneca, Cepheid, Konica—Minolta, Lilly, NextCure personal fees from Cell Signaling Technology, Danaher, Fluidigm, GSK, Merck, Monopteros, NanoString, Odonate, Paige. AI, Regeneron, Roche, Sanofi, Ventana and Verily, royalties from Rarecyte, all outside the submitted work.

CG reports grants and personal fees from NeraCare, Novartis, Roche, Sanofi, personal fees from Amgen, BMS, MSD, and Philogen, all outside the submitted work.

TE reports personal fees from Novartis, BMS, Almirall Hermal, CureVac, Sanofi, MSD, Pierre Fabre and institutional grants from MSD, Sanofi, BMS, Pfizer, GenenTech, Seagan, Regeneron all outside the submitted work.

IB reports having received speaker fees from Bayer, Pfizer, Takeda and AstraZeneca.

MR reports grants from AB Science, Abbott, AbbVie, Alcedis, Almirall Hermal, Amgen, Anaptys Bio, Argenx, AstraZeneca, Bayer, Biogen Idec, Boehringer Ingelheim, Bristol Myers Squibb, Celgene, CureVac, DelArrivo, Deutsche Forschungsgemeinschaft, Deutsche Krebshilfe, Dynavax Tech, Eli Lilly, Galderma, Genentech, GSK, Hoffmann La Roche, Hokusai, Idera Pharmaceuticals, Ilkos Therapeutic, Immatics biotechnologies, Incyte, Iovance Biotherapeutics, Janssen Cilag, Johnson & Johnson, LEO Pharma, Merck, MSD Sharp &Dohme, Novartis Pharmaceuticals, PellePharm, Pfizer, Philogen, Regeneron Pharmaceuticals, Sanofi Aventis, Schering Plough, Sun Pharma, Technische Universitat Dresden, Topaz Therapeutics, UCB, Universitatsklinik Essen, Universitatklinik Koln, Wilhelm Sander-Stiftung, 4SC.

The other authors report no potential conflicts of interest.
