# Supplementary material for: Deep learning-based scoring of tumour-infiltrating lymphocytes is prognostic in primary melanoma and predictive to PD-1 checkpoint inhibition in melanoma metastases
Source: eBioMedicine. 2023 Jun 7;93:104644. doi: 10.1016/j.ebiom.2023.104644 (PMC10363450; doi:10.1016/j.ebiom.2023.104644)
Supplement: Supplementary Figs. S1–S9 and Tables S1–S3 [file mmc1.docx]

# **Deep learning-based scoring of tumour-infiltrating lymphocytes is prognostic in primary melanoma and predictive to PD-1 checkpoint inhibition in melanoma metastases**

**Supplementary data**

a

**Cohort A**

Assessed for eligibility (n=1695)

Central Malignant Melanoma Registry in Germany: stage I and II cutaneous melanoma patients diagnosed between 2010-2018

Excluded (n=1192)

- No H&E slide available (n=1189)
- Age<18 years (n=3)

Enrolled to the study (n=503)

Excluded (n=77)

- Low tumor content (n=31)
- Bad quality of H&E slides (n=30)
- Extended necrosis (n=7)
- Extensive pigment incontinence (n=9)

Metastatic samples as matched pairs (n=89)

Primary melanoma samples (n=426)

- Stage IA: difficult to assess due to TD≤1 mm (n=105)

Fully analyzed:

- Stages IB, IIA, IIB, IIC (n=321)

Included to the study (n=426)

b

**Cohort B**

Assessed for eligibility (n=1137)

-Central Malignant Melanoma Registry in Germany: stage IV cutaneous melanoma patients diagnosed between 2010-2018

(n=1084)

-Dresden (n=38)

-St. Gallen (n=15)

Excluded (n=914)

- No H&E slides of metastases available (n=850)
- Age<18 years (n=2)
- No treatment-naïve metastatic sample (n=62)

Enrolled to the study (n=223)

Excluded (n=32)

- Low tumor content (n=6)
- Bad quality of slides (n=26)

Included to the study and analyzed (n=191)

**Supplementary Fig. 1**. **Flowchart showing the workflow of this retrospective study and how we included patients in the final analysis**. (a) Cohort A included patients diagnosed initially with stage I and II primary cutaneous melanoma. (b) Cohort B included patients diagnosed with stage III and IV cutaneous melanoma.


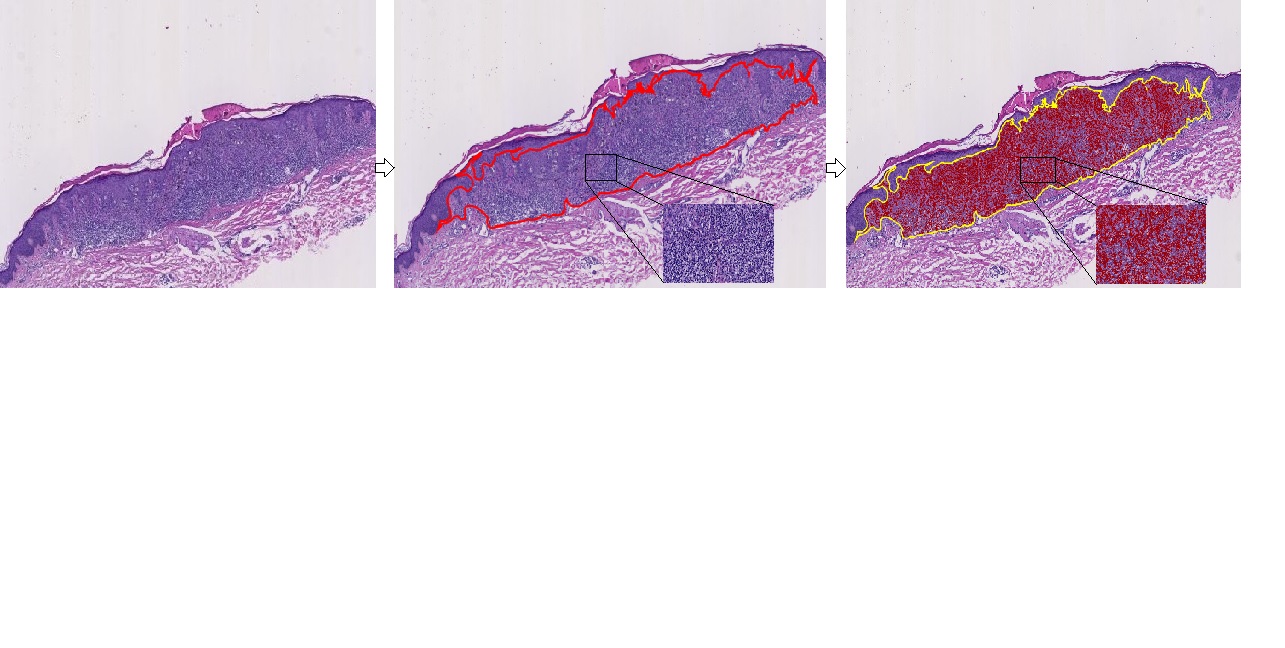
**Supplementary Fig. 2**. **Workflow of TIL quantification.** Firstly, we selected the tumour area, then *Qupath v.0.1.2* performed cell segmentation, and finally, the *NN192* classifier identified tumour cells and TILs. eTILs was manually calculated as [TILs/(TILs+tumour cells)]*100%.


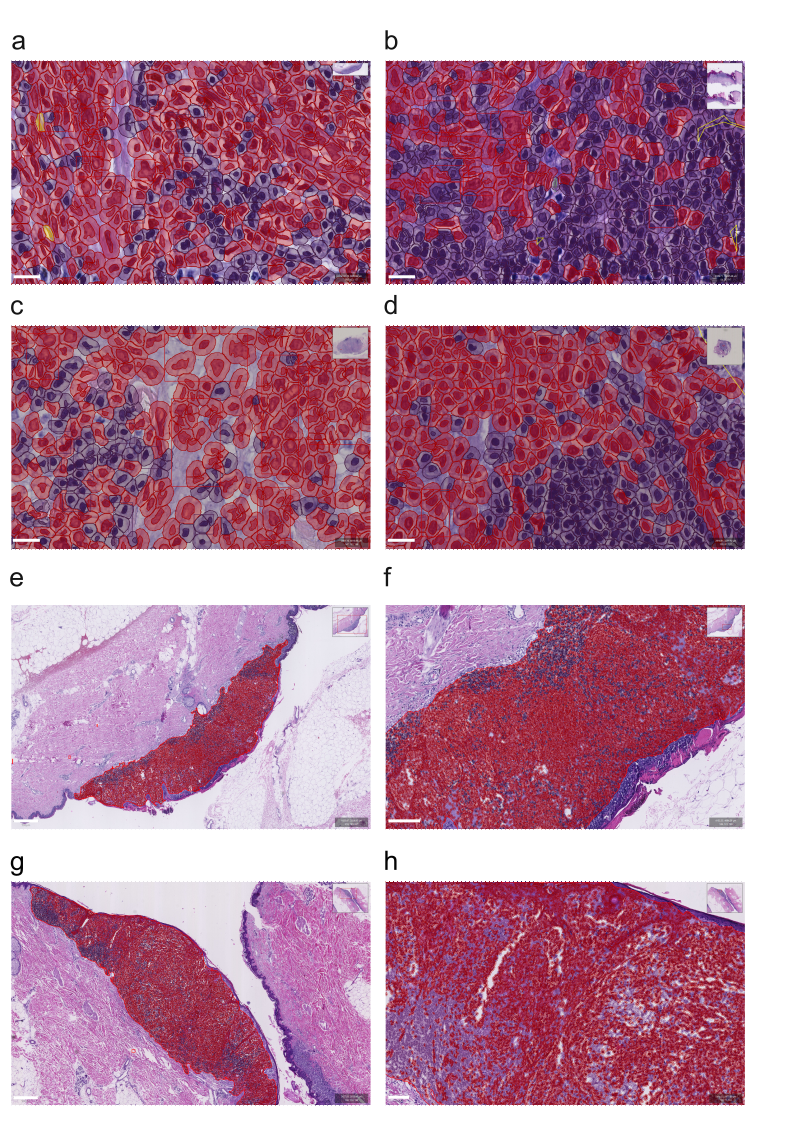


**Supplementary Fig. 3. Overview images of melanoma samples** (a, b) examples of primary melanoma samples where performance metrics of the classifier to identify TILs were calculated against visual inspection; scale bar: 20 μm; Rectangles represent the annotation through visual inspection; (c, d) examples of melanoma metastases where performance metrics of the classifier to identify TILs were calculated against visual inspection; Rectangles represent the annotation through visual inspection; (e, f) Images from the same melanoma at different magnification; scale bars: 500 μm (e); 200 μm (f); (g, h) Images from the same melanoma at different magnification; scale bars: 500 μm (g); 100 μm (h).

**Supplementary Table 1. Patient clinicopathological characteristics and median eTILs of primary tumours**

| **Characteristics** | **eTILs (%) of primary** |  |
| --- | --- | --- |
| n=321 | **Median [IQR]** | **p value** |
| **Age group at Dx (y)** |  | 0.216 |
| ≤65 | 22.51 [15.95-32.17] |  |
| >65 | 20.52 [14.41-32.32] |  |
| **Sex** |  | 0.418 |
| Female | 22.05 [14.84-32.17] |  |
| Male | 22.50 [14.78-32.10] |  |
| **Localization** |  | 0.732 |
| Head and neck | 21.87 [16.30-34.13] |  |
| Trunk | 22.28 [14.51-33.01] |  |
| Upper extr. | 21.57 [18.02-28.90] |  |
| Lower extr. | 21.94 [13.63-30.96] |  |
| **Histological subtype*** |  | **0.002** |
| SSM | 22.73 [15.86-31.56] |  |
| NM | 16.73 [10.80-24.44] |  |
| LMM | 25.80 [18.13-39.60] |  |
| ALM | 22.60 [14.71-33.22] |  |
| Unknown | 27.70 [20.41-35.09] |  |
| ***BRAF* V600 oncogenic variant** |  | 0.7305 |
| No | 20.11 [13.54-29.90] |  |
| Yes | 21.21 [12.61-34.63] |  |
| Unknown | 24.57 [16.31-33.57] |  |
| **Stage at Dx** |  | 0.482 |
| I | 23.29 [15.75-30.70] |  |
| II | 20.98 [13.86-33.01] |  |
| **Substage at Dx** |  | **<0.001** |
| IA | NA |  |
| IB | 23.29 [15.75-30.70] |  |
| IIA | 27.05 [17.40-37.53] |  |
| IIB | 20.97 [11.37-31.61] |  |
| IIC | 15.69 [ 9.50-20.54] |  |
| **Tumour thickness group (mm)** |  | **<0.001** |
| ≤1.0 | NA |  |
| 1.1 to 2.0 | 24.71 [16.23-34.62] |  |
| 2.1 to 4.0 | 22.61 [15.86-34.10] |  |
| >4.0 | 17.72 [10.21-21.46] |  |
| **Ulceration** |  | 0.952 |
| No | 23.26 [15.75-32.21] |  |
| Yes | 20.33 [13.23-31.13] |  |
| **Regression*** |  | **0.009** |
| No | 21.48 [14.54-30.83] |  |
| Yes | 27.10 [18.37-36.53] |  |
| Unknown | 20.60 [13.86-29.12] |  |

Values are reported as a median and interquartile range [IQR] and compared using the Mann-Whitney test for two variables and Kruskal Wallis for more than two comparisons; SSM, *S*uperficial spreading melanoma; NM, Nodular melanoma; LMM, Lentigo malignant melanoma; ALM, Acrolentiginous melanoma; y, years; extr., extremities; Dx, diagnosis; *Patients for whom information was not available are not considered for comparisons


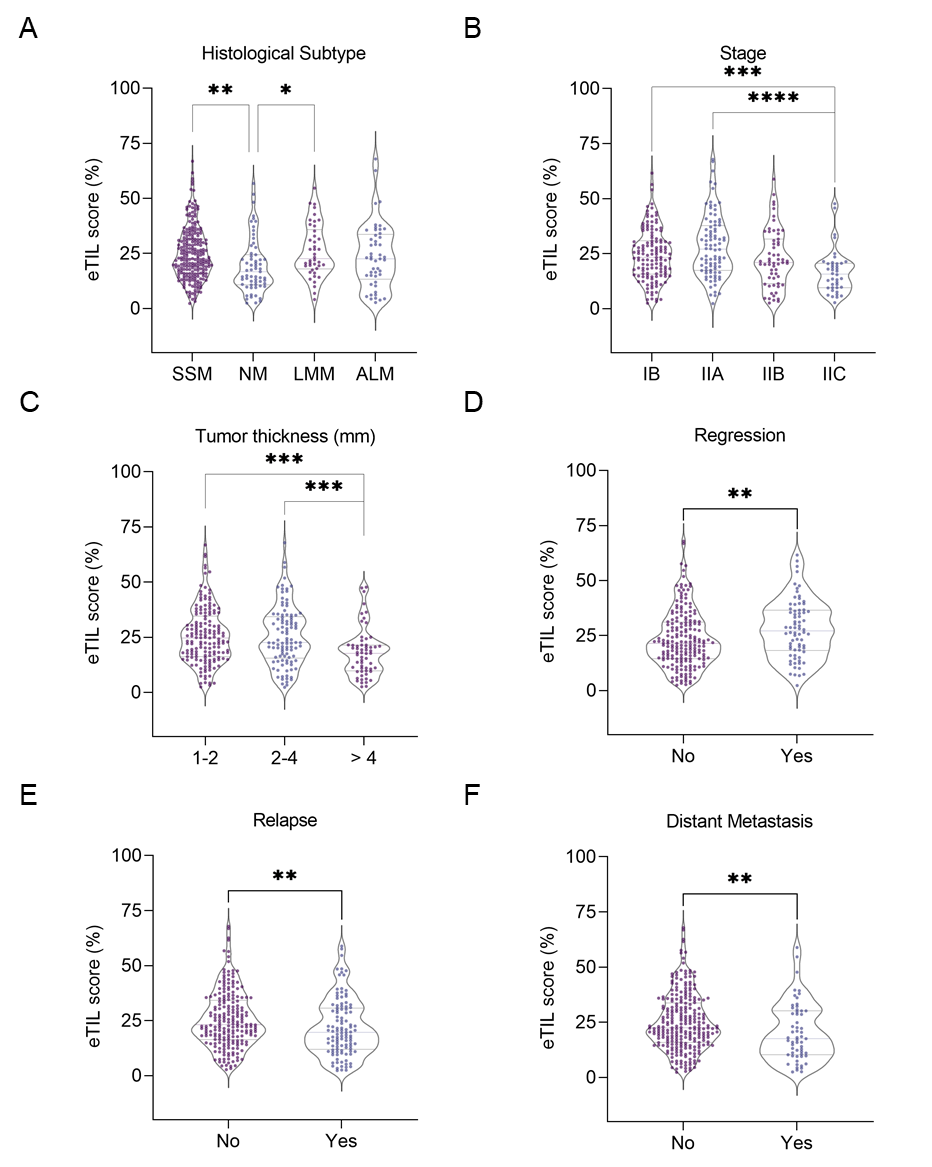


d

c

b

a

**Supplementary Fig. 4**. **Violin plots showing the eTILs of primary tumours in relation to patient characteristics.** (a-d) Median, 25, and 75 percentiles are depicted; median eTILs were compared using compared using the Mann-Whitney U test for two variables and Kruskal Wallis test for more than two comparisons; *p<0.05, **p<0.01, ***p<0.001, ****p<0.0001.

**
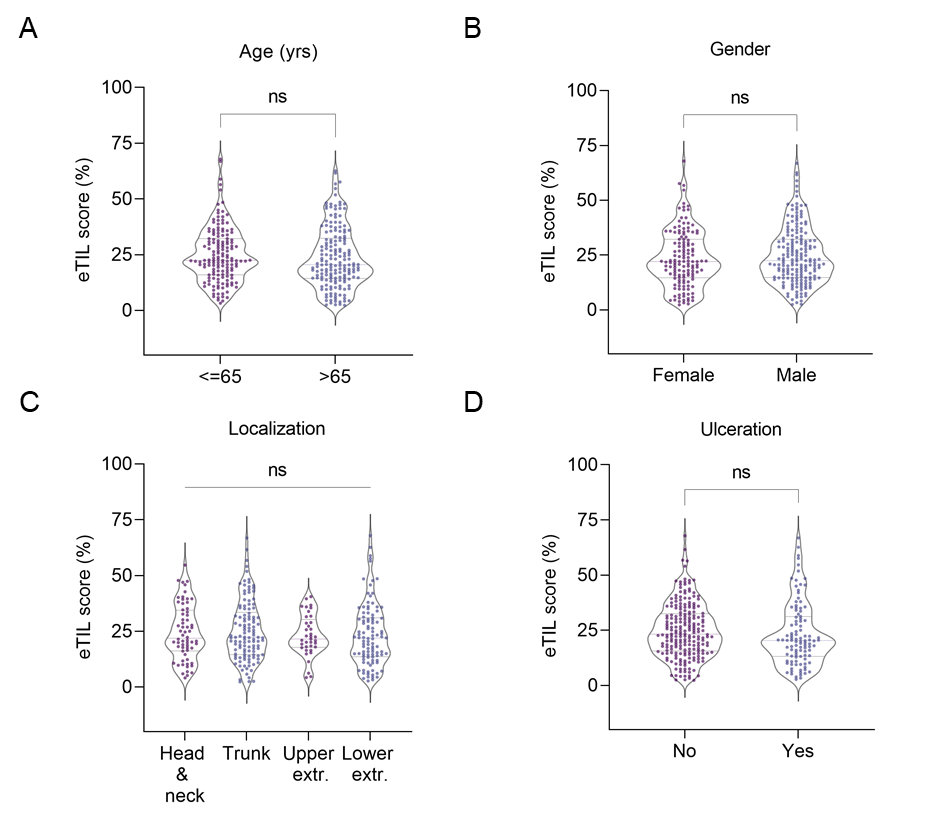
**

Sex

b

d

c

a

e

**Supplementary Fig. 5**. **Violin plots showing the eTILs of primary tumours in relation to patient characteristics**. (a-e) Median eTILs were compared using the Mann-Whitney U test for two variables and Kruskal Wallis test for more than two comparisons; yrs, years; extr., extremities Median, 25 and 75 percentiles are depicted; ns=not significant.

a


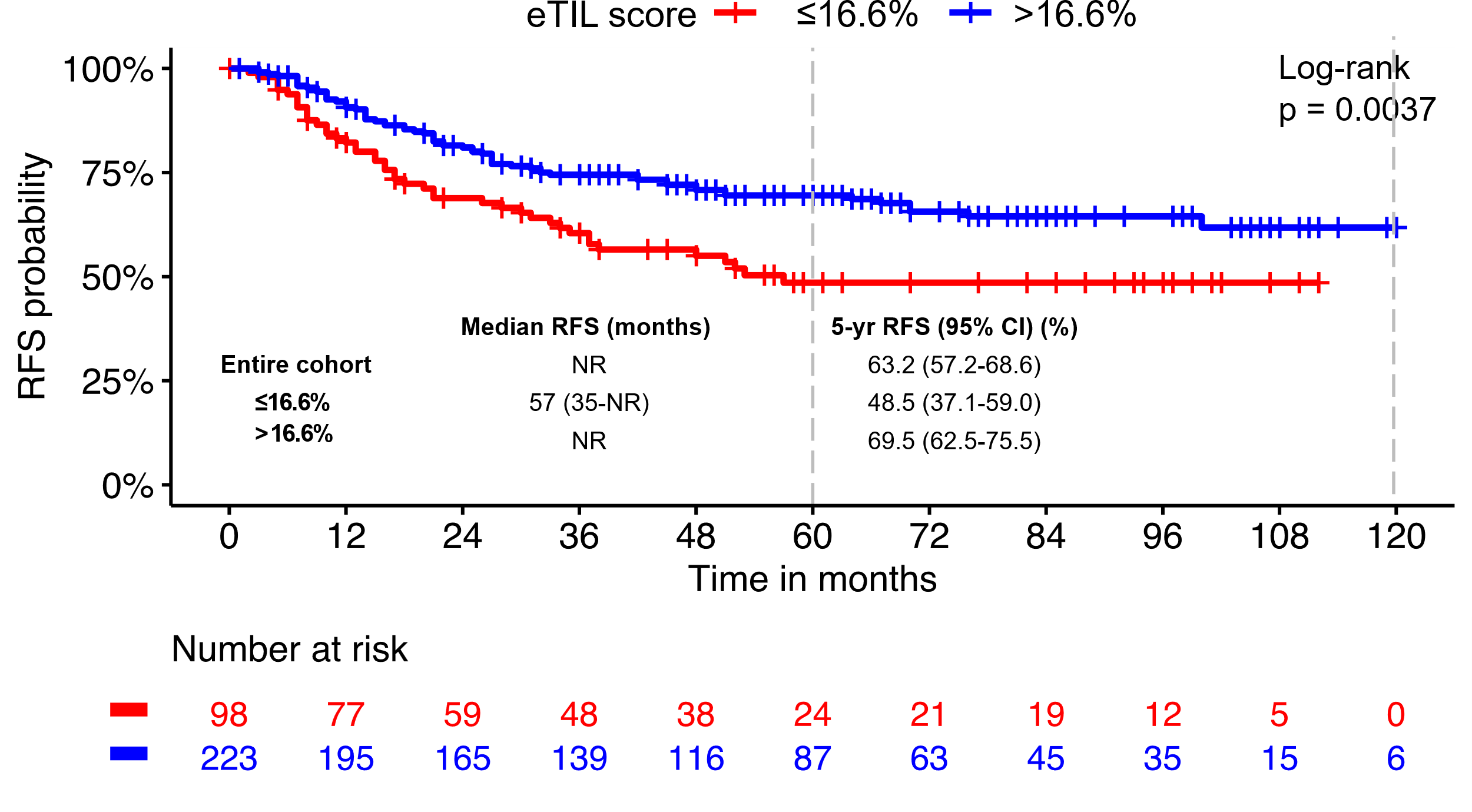


b


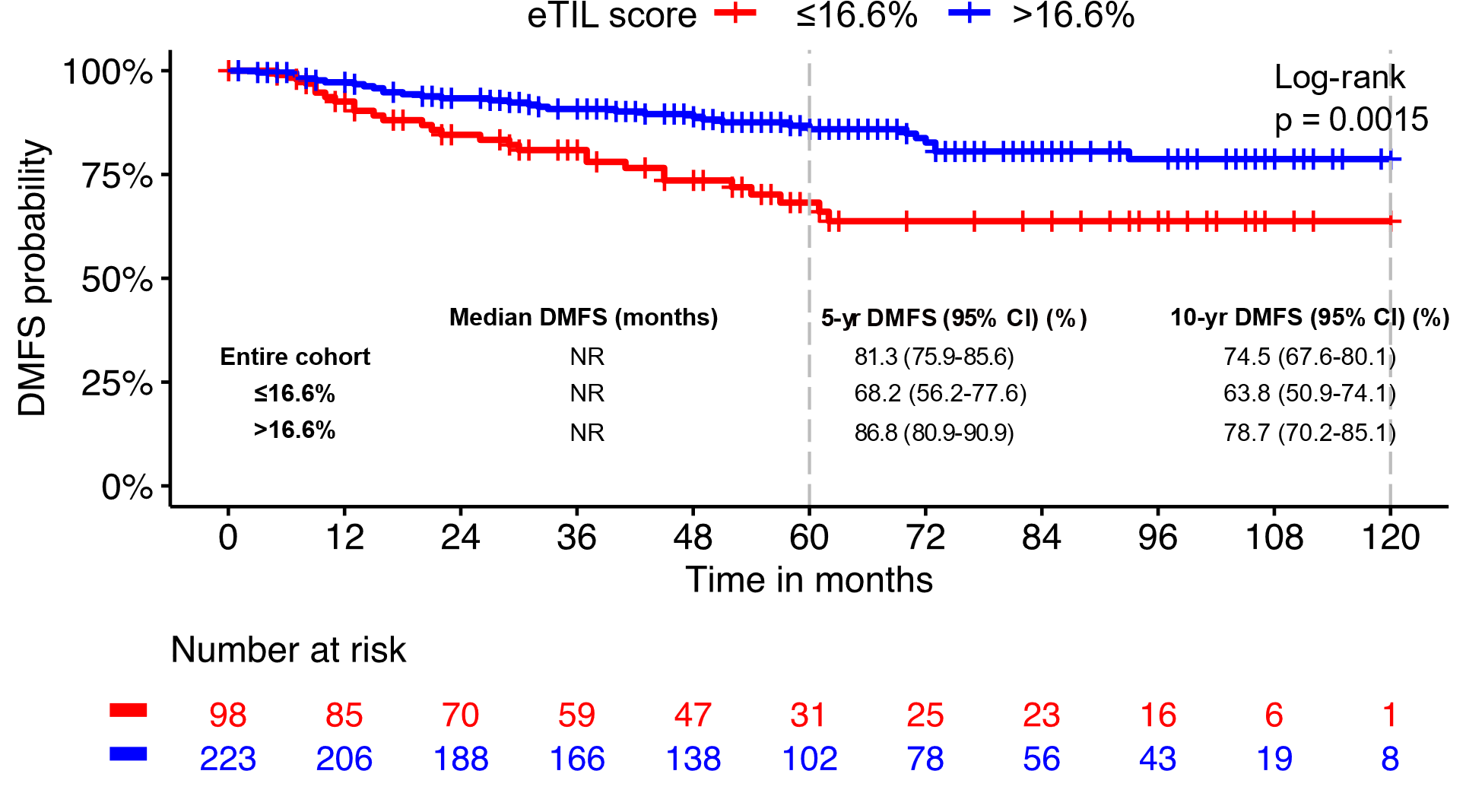


c

**
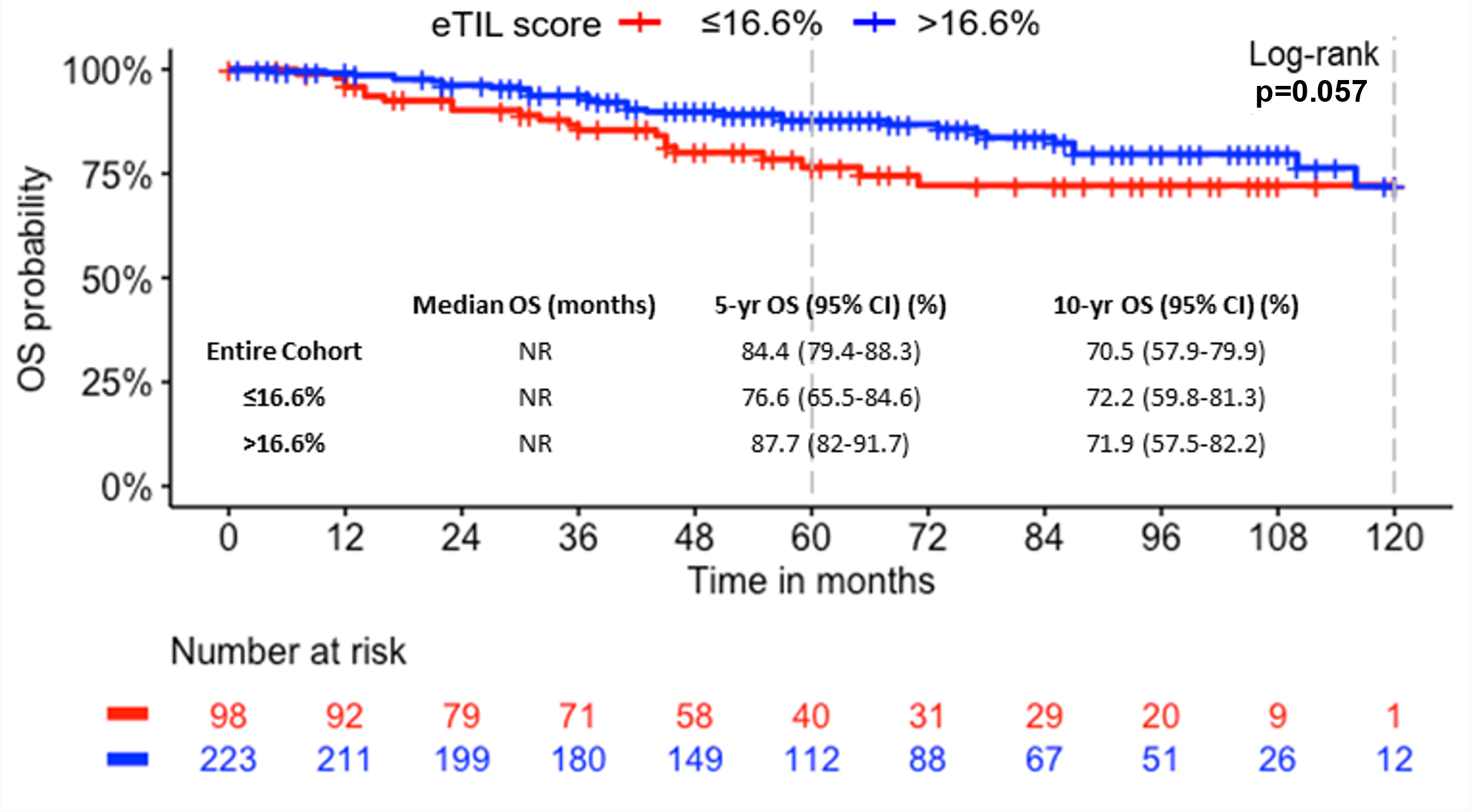
**

**Supplementary Fig. 6** **Kaplan-Meier curves of stage I/II melanomas with 10-year follow-up.** (a) RFS analysis for the 10-year follow-up after initial melanoma diagnosis for the 321 stage IB to IIC patients according to the eTILs group using the cut-off of 16.6%. It shows the Kaplan-Meier curves for RFS and the number of melanoma patients at risk at specific time points starting from primary melanoma diagnosis. (b) DMFS analysis for the 10-year follow-up after initial melanoma diagnosis for the 321 stage IB to IIC patients according to the eTILs group using the cut-off of 16.6%. It shows the Kaplan-Meier curves for DMFS and the number of melanoma patients at risk at specific time points starting from primary melanoma diagnosis. (c) OS analysis for the 10-year follow-up after initial melanoma diagnosis for the 321 stage IB to IIC patients according to the eTILs group using the cut-off of 16.6%. It shows the Kaplan-Meier curves for OS and the number of melanoma patients at risk at specific time points starting from primary melanoma diagnosis.

**Supplementary Table 2. Clinicopathological characteristics of patients from whom a metastatic sample was assessed (n=191)**

| Characteristics | Adjuvant anti-PD-1, N = 21*^1^* | Anti-CTLA-4, N = 7*^1^* | Non-adjuvant anti-PD-1, N = 101*^1^* | TT non adjuvant, N = 32*^1^* | No systemic therapy, N = 30*^1^* |
| --- | --- | --- | --- | --- | --- |
|  | N (%) | N (%) | N (%) | N (%) | N (%) |
| Matched |  |  |  |  |  |
| Matched | 15 (71) | 6 (86) | 31 (31) | 7 (22) | 30 (100) |
| No matched | 6 (29) | 1 (14) | 70 (69) | 25 (78) | 0 (0) |
| Cohort |  |  |  |  |  |
| Dresden | - | - | 4 (4.0) | - | - |
| St. Gallen | - | - | 12 (12) | - | - |
| Tuebingen | 21 (100) | 7 (100) | 85 (84) | 32 (100) | 30 (100) |
| eTILs | 12 [9-22] | 16 [9-19] | 13 [8-21] | 14 [7-26] | 10 [7- 18] |
| Age at therapy (yrs),  median [IQR] | 66 [56-77] | 72 [70-78] | 68 [59-77] | 60 [50-67] | NA |
| Age at therapy; yrs |  |  |  |  |  |
| ≤65 | 10 (49) | 1 (14,3) | 41 (40.6) | 23 (71.9) | NA |
| >65 | 11 (51) | 6 (85.7) | 60 (59.4) | 9 (29.1) | NA |
| Sex |  |  |  |  |  |
| Female | 10 (48) | 4 (57) | 37 (37) | 12 (38) | 19 (63) |
| Male | 11 (52) | 3 (43) | 64 (63) | 20 (62) | 11 (37) |
| 1st therapy after excision |  |  |  |  |  |
| Adjuvant anti-PD-1 | 21 (100) | - | - | - | - |
| Ipilimumab | - | 7 (100) | - | - | - |
| Anti-PD-1 monotherapy | - | - | 59 (59) | - | - |
| Nivolumab+Ipilimumab | - | - | 42 (41) | - | - |
| Dabrafenib | - | - | - | 4 (12) | - |
| Dabrafenib+Trametinib | - | - | - | 7 (22) | - |
| Vemurafenib | - | - | - | 9 (28) | - |
| Vemurafenib+Cobimetinib | - | - | - | 12 (38) | -- |
| No systemic therapy | - | - | - | - | 30 (100) |
| Stage at therapy |  |  |  |  |  |
| IIIA | 1 (4.8) | - | 1 (1.0) | - | 1 (3.3) |
| IIIB | 4 (19) | 1 (14) | 1 (1.0) | - | - |
| IIIC | 8 (38) | 1 (14) | 7 (6.9) | 4 (12) | 1 (3.3) |
| IIID | 2 (9.5) | - | - | - | - |
| IV-M1a | 1 (4.8) | 1 (14) | 10 (9.9) | 1 (3.1) | 1 (3.3) |
| IV-M1b | 1 (4.8) | 1 (14) | 18 (18) | 5 (16) | - |
| IV-M1c | 3 (14) | 2 (29) | 46 (46) | 14 (44) | 2 (6.7) |
| IV-M1d | 1 (4.8) | 1 (14) | 18 (18) | 8 (25) | - |
| NA | - | - | - | - | 25 (83) |
| *BRAF* mutation |  |  |  |  |  |
| Mutated | 5 (24) | 1 (14) | 27 (27) | 32 (100) | 4 (13) |
| Wt | 15 (71) | 5 (71) | 72 (71) | - | 13 (43) |
| Unknown | 1 (4.8) | 1 (14) | 2 (2.0) | - | 13 (43) |
| Brain Metastases |  |  |  |  |  |
| Metastases | 1 (4.8) | 1 (14) | 18 (18) | 8 (25) | - |
| No metastases | 20 (95) | 6 (86) | 83(82) | 24 (75) | 30 (100) |
| LDH at therapy start (U/l),  median [IQR] | 208[180-246] | 185[171-227] | 223[184-300] | 231[199-292] | NA |
| NA | 3 | 0 | 0 | 0 | 30 |
| LDH at therapy start; U/l |  |  |  |  |  |
| ≤250 | 14 (77.8) | 7 (100) | 63 (62.4) | 20 (62.5) | NA |
| >250 | 4 (22.2) | 0 | 38 (37.6) | 12 (37.5) | NA |

Values are reported as counts (n) and percentages (%) for discrete values and as a median and interquartile range [IQR] for continuous values.

a b

c d

e f


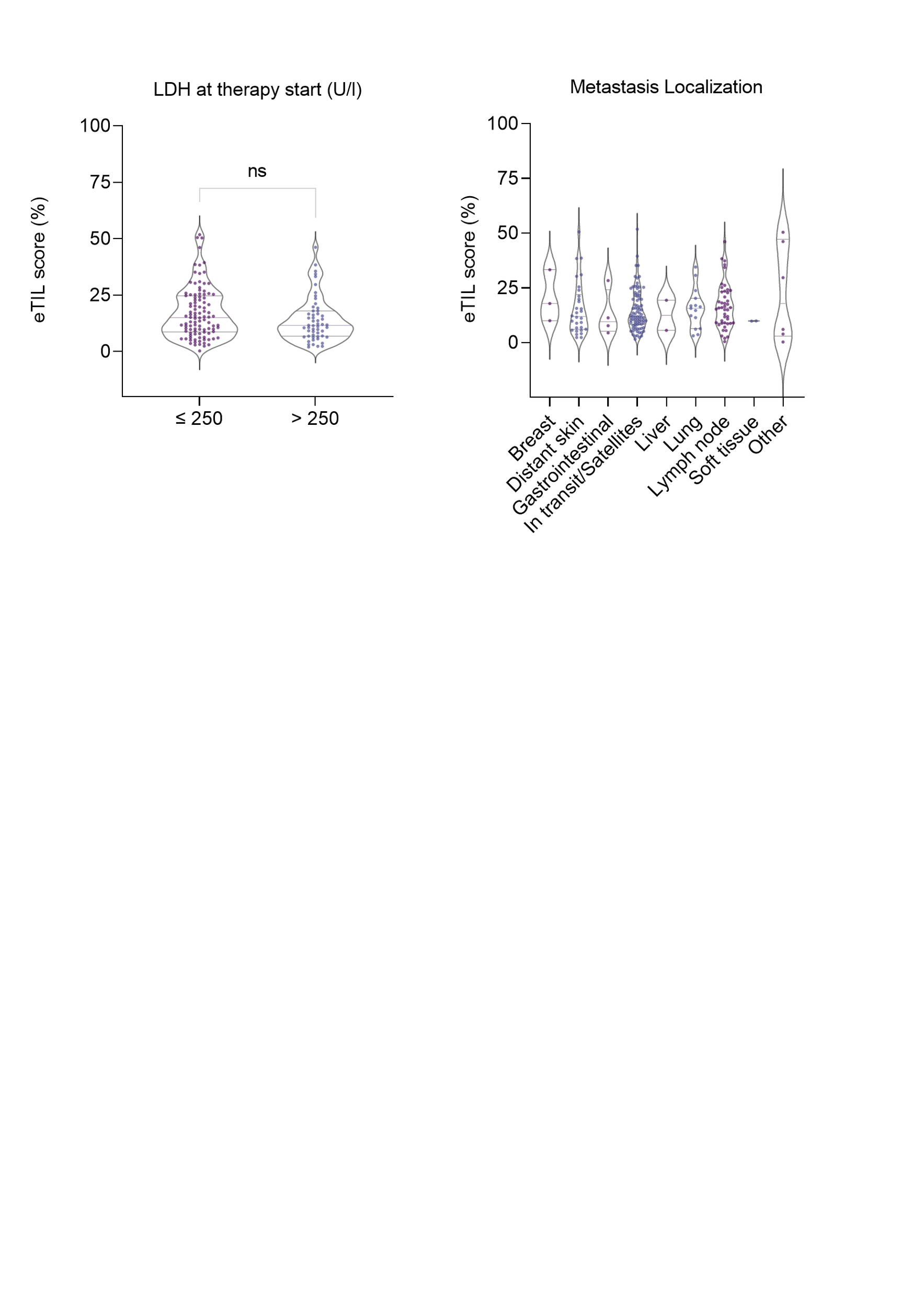


**Supplementary Fig. 7**. **Violin plots showing the eTILs of metastases in relation to patient characteristics at the therapy start of first-line anti-PD-1 and localization of metastasis.** (a-f) Median, 25, and 75 percentiles are depicted; median eTILs were compared using the Mann-Whitney U test for two variables; *p<0.05, **p<0.01, ***p<0.001, ****p<0.0001; ns=not significant.

**Supplementary Table 3. Clinicopathological characteristics of patients who were included in the matched pair analysis**

| Features | n=89 (100%) |
| --- | --- |
|  | **n (%)** |
| Metastatic sample, n (%) |  |
| Distant lymph nodes | 10 (11.2) |
| Regional lymph nodes | 28 (31.5) |
| In-transit | 38 (42.7) |
| Distant visceral or skin metastases | 13 (14.6) |
| Age at Dx (y), median [IQR} | 76 [67-83] |
| Age group at Dx (y), n (%) |  |
| ≤65 | 32 (36) |
| >65 | 57 (64) |
| Sex, n (%) |  |
| Female | 45 (50.6) |
| Male | 44 (49.4) |
| Localization of primary tumour, n (%) |  |
| Head and neck | 19 (21.3) |
| Trunk | 25 (28.1) |
| Upper extr. | 9 (10.1) |
| Lower extr. | 36 (40.5) |
| Histological subtype, n (%) |  |
| SSM | 33 (37.1) |
| NM | 24 (27) |
| LMM | 6 (6.7) |
| ALM | 17 (19.1) |
| Unknown | 9 (10.1) |
| *BRAF* oncogenic variant, n (%) |  |
| Mutated | 23 (25.8) |
| Wt | 52 (58.4) |
| Unknown | 14 (15.7) |
| Stage at first Dx, n (%) |  |
| I | 27 (30.3) |
| II | 62 (69.7) |
| Substage at first Dx, n (%) |  |
| IA | NA |
| IB | 27 (30.3) |
| IIA | 26 (29.2) |
| IIB | 20 (22.5) |
| IIC | 16 (18.0) |
| 1st therapy after excision, n (%) |  |
| Adjuvant anti-PD-1 | 15 (16.9) |
| Ipilimumab | 6 (6.7) |
| Anti-PD-1 therapy | 31 (34.8) |
| Targeted therapy | 7 (7.9) |
| No systemic therapy | 30 (33.7) |

Values are reported as counts (n) and percentages (%) for discrete values and as a median and interquartile range [IQR] for continuous values; y, years; dx, diagnosis

a


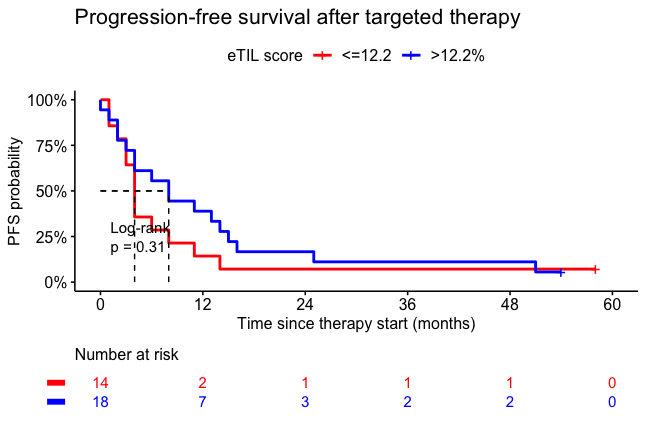


b


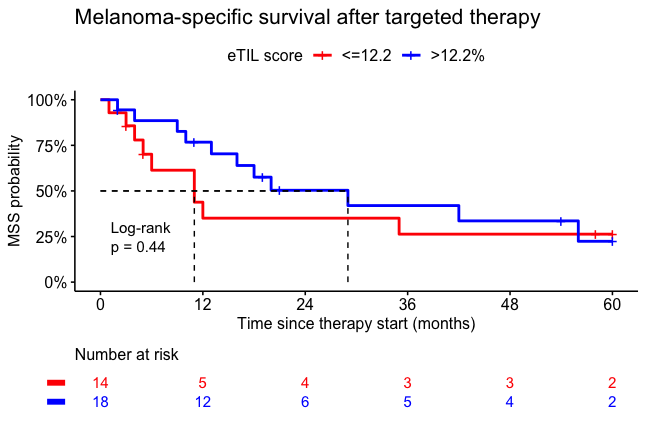


c


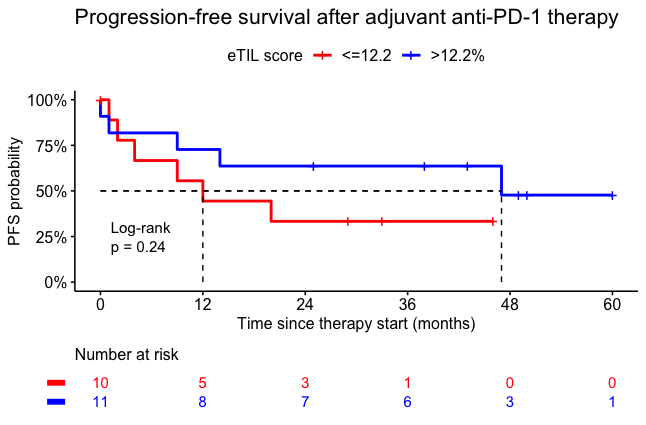


d


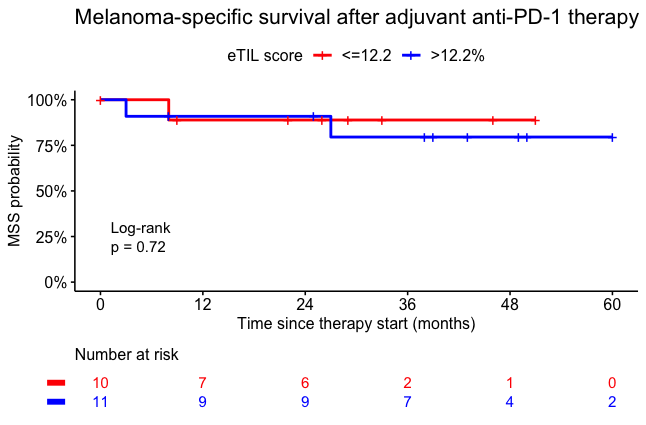


**Supplementary Fig. 8**. **Kaplan-Meier curves of patients receiving targeted therapy or adjuvant first-line anti-PD-1.** (a) Progression-free survival (PFS) analysis for the 5-year follow-up after targeted therapy start for the 32 stage III/IV patients according to eTILs group using the cut-off 12.2%; (b) Melanoma-specific survival (MSS) analysis for the 5-year follow-up period after targeted therapy start for the 32 stage III/IV patients according to eTILs group using the cut-off 12.2%; (c) Progression-free survival (PFS) analysis for the 5-year follow-up period after adjuvant anti-PD-1-based therapy start for the 21 stage III/IV patients according to eTILs group using the cut-off 12.2%; (d). Melanoma-specific survival (MSS) analysis for the 5-year follow-up period after adjuvant anti-PD-1-based therapy start for the 21 stage III/IV patients according to eTILs group using the cut-off 12.2%.


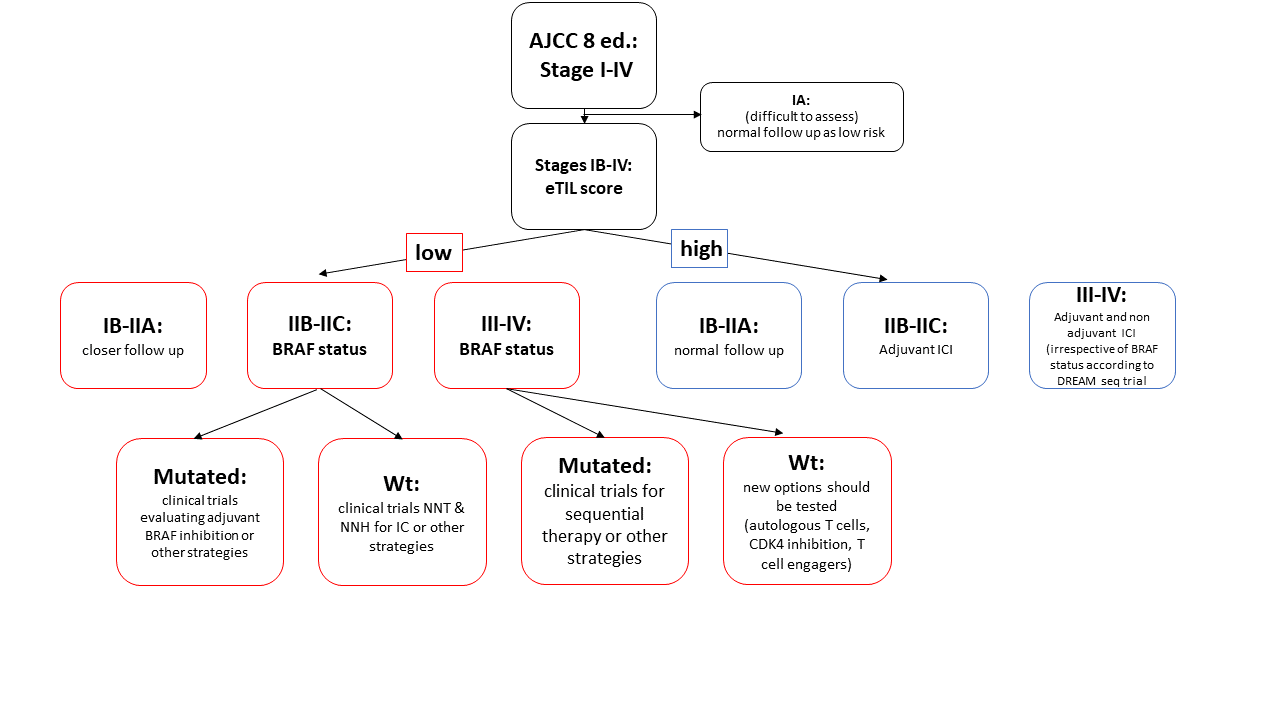


**Supplementary Fig. 9**. Schema showing how eTILs could be applied in the clinic regarding therapeutic decisions.
